# CHIMERA repetitive mild traumatic brain injury induces chronic behavioural and neuropathological phenotypes in wild-type and APP/PS1 mice

**DOI:** 10.1186/s13195-018-0461-0

**Published:** 2019-01-12

**Authors:** Wai Hang Cheng, Kris M. Martens, Asma Bashir, Honor Cheung, Sophie Stukas, Ebrima Gibbs, Dhananjay R. Namjoshi, Emily B. Button, Anna Wilkinson, Carlos J. Barron, Neil R. Cashman, Peter A. Cripton, Cheryl L. Wellington

**Affiliations:** 1 Department of Pathology and Laboratory Medicine, Djavad Mowafaghian Centre for Brain Health, University of British Columbia, 2215 Wesbrook Mall, Vancouver, BC V6T 1Z3 Canada; 2 Department of Neurology, Djavad Mowafaghian Centre for Brain Health, University of British Columbia, 2215 Wesbrook Mall, Vancouver, BC V6T 1Z3 Canada; 3 Department of Mechanical Engineering, International Collaboration on Repair Discoveries, University of British Columbia, 6250 Applied Sciences Lane, Vancouver, BC V6T 1Z4 Canada

**Keywords:** Traumatic brain injury, CHIMERA, Alzheimer disease mice, Post-traumatic stress disorder, Spatial memory, Neuroinflammation, Aβ metabolism

## Abstract

**Background:**

The annual incidence of traumatic brain injury (TBI) in the United States is over 2.5 million, with approximately 3–5 million people living with chronic sequelae. Compared with moderate-severe TBI, the long-term effects of mild TBI (mTBI) are less understood but important to address, particularly for contact sport athletes and military personnel who have high mTBI exposure. The purpose of this study was to determine the behavioural and neuropathological phenotypes induced by the Closed-Head Impact Model of Engineered Rotational Acceleration (CHIMERA) model of mTBI in both wild-type (WT) and APP/PS1 mice up to 8 months post-injury.

**Methods:**

Male WT and APP/PS1 littermates were randomized to sham or repetitive mild TBI (rmTBI; 2 × 0.5 J impacts 24 h apart) groups at 5.7 months of age. Animals were assessed up to 8 months post-injury for acute neurological deficits using the loss of righting reflex (LRR) and Neurological Severity Score (NSS) tasks, and chronic behavioural changes using the passive avoidance (PA), Barnes maze (BM), elevated plus maze (EPM) and rotarod (RR) tasks. Neuropathological assessments included white matter damage; grey matter inflammation; and measures of Aβ levels, deposition, and aducanumab binding activity.

**Results:**

The very mild CHIMERA rmTBI conditions used here produced no significant acute neurological or motor deficits in WT and APP/PS1 mice, but they profoundly inhibited extinction of fear memory specifically in APP/PS1 mice over the 8-month assessment period. Spatial learning and memory were affected by both injury and genotype. Anxiety and risk-taking behaviour were affected by injury but not genotype. CHIMERA rmTBI induced chronic white matter microgliosis, axonal injury and astrogliosis independent of genotype in the optic tract but not the corpus callosum, and it altered microgliosis in APP/PS1 amygdala and hippocampus. Finally, rmTBI did not alter long-term tau, Aβ or amyloid levels, but it increased aducanumab binding activity.

**Conclusions:**

CHIMERA is a useful model to investigate the chronic consequences of rmTBI, including behavioural abnormalities consistent with features of post-traumatic stress disorder and inflammation of both white and grey matter. The presence of human Aβ greatly modified extinction of fear memory after rmTBI.

**Electronic supplementary material:**

The online version of this article (10.1186/s13195-018-0461-0) contains supplementary material, which is available to authorized users.

## Background

Traumatic brain injury (TBI) is defined as “an alteration in brain function, or other evidence of brain pathology, caused by an external force” [61]. In the United States, the annual incidence of TBI is over 2.5 million [14], and over 3–5 million persons are living with chronic sequelae of TBI [20, 90]. The Glasgow Coma Scale (GCS) is used to classify TBI severity into categories of mild (mTBI: GCS 13–15), moderate (GCS 8–12), or severe (GCS 3–7) [13, 89]. Moderate and severe TBI are associated with high rates of disability or death within 1 year of injury [40] and a significantly increased risk (approximately 1.5-fold) of dementia, including Alzheimer disease (AD) [26, 30, 65]. Long-term survivors of moderate and severe TBI also have numerous psychiatric symptoms, such as anxiety and depression, as well as cognitive impairments in memory and processing speed [46]. These symptoms persist both at the recovery plateau (6–18 months post-injury) and thereafter [80].

Less well understood are the chronic effects of mTBI, which comprises at least 80% of all TBI cases [12, 24, 51, 73]. Between 10% and 55% of patients with mTBI report persistent symptoms from 3 months to 1 year after injury [58, 69]. The term *post-concussion syndrome* (PCS) describes the cognitive, somatic, sleep and other changes that occur as a consequence of mTBI and persist for at least 3 months [5]. PCS symptoms include irritability, mood disturbances, concentration difficulties, depression, and anxiety disorders [5, 45]. Post-traumatic stress disorder (PTSD), characterized by intrusive thoughts and deficient fear memory extinction [48, 92], is another frequent occurrence among patients with mTBI, particularly in the military setting [34]. Overall, subjects with mTBI have an elevated risk for any psychiatric illness in 6 months post-injury (OR = 2.8 or 1.6 for subjects without or with a prior history of psychiatric illness, respectively) [23]. Repetitive exposure to mTBI is also linked to the development of a neurodegenerative condition called chronic traumatic encephalopathy (CTE), which is of particularly high relevance to athletes in contact sports and in military personnel [59, 77] because both groups may be exposed to a large number of mTBIs during their careers. CTE is a neuropathological disorder defined by perivascular tau deposits primarily in sulcal depths [60]; however, behaviourally, CTE is associated with irritability, impulsivity, aggression, suicidality, and memory loss [59].

In addition to chronic behavioural changes, neuroinflammatory changes are also common long-term features of TBI. Immunohistological examination (using CR3/43 and CD68) and imaging studies (using [^11^C](R)PK11195) of brains from patients with moderate or severe TBI have shown that chronic neuroinflammatory changes may persist for months to up to 10 years after TBI [41, 75]. Chronic inflammation in white matter and the thalamus are associated with white matter degeneration [41] and cognitive impairment [75], respectively. In a clinical study involving 66 deceased football athletes with CTE, the number of years playing football was associated with microglia activation and dementia status [18].

Neuropathological changes are common after TBI. Amyloid-β (Aβ) deposits are found in 30% of patients who die of severe TBI in the acute phase [33, 37, 42, 44, 78]. Recent studies using positron emission tomography-computed tomography (PET-CT) confirm increased [^11^C] Pittsburgh compound B ([^11^C]PiB) binding in the living brain during the acute phase after moderate or severe TBI [35]. Long-term Aβ changes after a single moderate or severe TBI, however, are less conclusive, with studies showing either no change in amyloid [16], region-specific changes in amyloid [31], or altered amyloid structure but not density [43]. In CTE brains, amyloid deposits are observed only in up to 50% of cases [91].

Animal model studies are useful to investigate the behavioural and neuropathological consequences of mTBI. We recently established Closed-Head Impact Model of Engineered Rotational Acceleration (CHIMERA) as a biomechanically and clinically relevant mTBI model [66, 68]. CHIMERA induces TBI by delivering highly reproducible impacts to a freely moving head, enabling integrated analysis of head kinematics with biological outcomes. CHIMERA reliably produces diffuse axonal injury throughout the brain and can differentiate between concussive and subconcussive injuries [66]. We recently described acute outcomes of CHIMERA repetitive mTBI (rmTBI) in male APP/PS1 mice, a well-established model of AD amyloidosis [17]. In this study, animals received two mild impacts at 0.5 J energy (2 × 0.5 J), 24 h apart, at either 5.5 or 13.5 months of age and were assessed from 6 h up to 14 days post-injury. We observed that age at injury, in addition to genetic predisposition to amyloid, modulates several acute outcomes, including Aβ deposition, neuroinflammation, and axonal injury responses. In the present study, we used a similar study design to deliver 2 × 0.5 J CHIMERA impacts to both WT and APP/PS1 littermates at 5.7 months of age, and we report the long-term behavioural and neuropathological effects up to 8 months post-injury.

## Methods

### Animals and CHIMERA procedure

Male APP/PS1 transgenic mice [B6C3-Tg(APPswe,PSEN1dE9)85Dbo/Mmjax] and WT non-transgenic littermate controls were used in this study (total *N* = 46). The colony was maintained on an F1 hybrid C57BL/6×C3H background to control for genetic admixture between APP/PS1 and non-transgenic WT littermates. After weaning, mice were housed with environmental enrichment on a 12-h/12-h reverse light cycle and were fed the 2918 Teklad global 18% protein rodent diet (Envigo, Madison, WI, USA) and autoclaved reverse osmosis water ad libitum.

Mice were 174 ± 18 days old (mean ± SD, approximately 5.7 months, at the early stages of amyloid deposition) when they were randomized to sham or CHIMERA rmTBI groups. Mice were given 0.5 ml of 0.9% saline for fluid supplementation and 1 mg/kg meloxicam for analgesia immediately before the CHIMERA procedure. Anaesthesia was induced with 5% isoflurane in 0.8 L/min oxygen and maintained at 4.0–4.5%. Anaesthetized mice were restrained in the supine position on the CHIMERA device such that their heads were free to move and rested at an angle of 145 degrees relative to the body. Two mild impacts (0.5 J impact energy) were delivered 24 h apart using a pneumatically driven 50-g piston with a 5-mm tip enclosed by a rubber cap. The piston impacted the midline parietal region, perpendicular to the long axis of the head, inducing the head to rotate in the sagittal plane. Sham-operated mice experienced NaCl, meloxicam, anaesthesia, restraint, anaesthesia, and meloxicam, but no impact. Over the 8 months of this study, a total of ten mice died prematurely. Of these, three cases occurred within 1–2 months after sham/TBI, three cases occurred after 3–4 months, and four cases occurred after 6–7 months. The original numbers of mice for all four groups (WT-Sham, WT-TBI, APP/PS1-Sham, APP/PS1-TBI) at the start of the study were 13, 11, 10, and 12, respectively. The numbers that completed the study were 12, 10, 6, and 8, respectively. For behavioural analysis, all data points (including those generated by mice that eventually died prematurely) were included. For histological analysis, only mice that survived up to the 8-month time point were included, and all available brains were analysed. The study design is summarized in Additional file 1.

### Behaviour

Behavioural analyses, including loss of righting reflex (LRR), Neurological Severity Score (NSS), rotarod (RR), passive avoidance (PA), Barnes maze (BM) and elevated plus maze (EPM), were performed as described previously [17, 68]. LRR was assessed immediately after the first and second injuries. NSS was assessed at baseline, 1 h, and day 1 (D1), D2, and D7 after injury. Other behaviours were assessed at both acute and chronic time points. Specifically, RR performance was assessed at baseline, D1, D2, D7 and D14 post-injury and month 1 (1 M), 2 M, 3 M, 5 M, 7 M, and 8 M post-injury using two consecutive testing days as the chronic time points, using a rotarod device from Ugo Basile (Collegeville, PA, USA). The EPM task was conducted on D7 and D10 and at 1 M, 2 M, 3 M, 6 M, 7 M, and 8 M post-injury using a homemade maze.

The PA task was performed using the Shuttle Box Avoidance Chamber from Med Associates Inc. (St. Albans, VT, USA). On shock days, mice were placed in a brightly illuminated light chamber. Once the mouse moved into the dark chamber, the guillotine door was shut and a mild foot shock (0.3 mA, 2 s) was delivered. On testing days, the mice were put in the same light chamber, but no shock was delivered if they moved to the dark chamber. PA duration is defined as the time that mice avoided entering the dark chamber (maximum 300 s). Shock 1 was performed at D6 post-injury, followed by testing days on D7, D8, and D9 and at 1 M, 2 M, and 3 M post-injury. After a 3-month resting period, at 6 months post-injury, the mice were subjected to Shock 2 and assessed for 3 consecutive days and again at 7 M and 8 M post-injury. The cumulative fear response was calculated from each mouse as AUC of the testing phase using the linear trapezoid rule [29]. The extinction portion of the PA curves from each mouse were modelled as forgetting curves using the Ebbinghaus savings function M = θt^-ψ^ [93, 94], where θ is initial memory and ψ is the rate of extinction. M_1_ (memory at D1 post-shock) was calculated by inputting *t* = 1.

The BM was purchased from Stoelting (Wood Dale, IL, USA). Acquisition trials of 90 s were conducted on D14, D15, D16, D17, and D18 post-injury; 30 s probe trials were conducted on D19 and 2 M, 6 M, and 8 M post-injury; and reverse trials were conducted the day after each probe trial. The spatial strategy used to find the BM hideout was classified using an automatic algorithm [38], based on the navigation plots of acquisition trials exported from Any-maze software (Stoelting). A cognitive score was calculated based on the weights of each strategy as reported [38]. The maze strategies from best to worst, and their associated scores, are Direct (1), Corrected (0.75), Long Corrected (0.5), Focus Search (0.5), Serial (0.25), and Random (0).

### Tissue collection, IHC and image analysis

Animals were killed with 150 mg/kg ketamine (Zoetis, Florham Park, NJ, USA) and 20 mg/kg xylazine (Bayer, Whippany, NJ, USA) at 8 M post-injury and perfused with 50 ml of ice-cold heparinized PBS (5 USP units/ml). Hemibrains were fixed in 4% paraformaldehyde for 2 days and cryoprotected with 30% sucrose for 2 days, after which 40-μm-thick coronal sections were cut using a cryotome (Leica Microsystems, Buffalo Grove, IL, USA). IHC was performed as described previously [17, 66, 68]. For the microglial marker Iba1, sections were quenched with hydrogen peroxide for 10 min, blocked with 5% bovine serum albumin, and incubated with primary antibodies overnight at 4 °C. Sections were then incubated with biotin-conjugated secondary antibodies (1:1000), then with ABC reagent (1:400; Vector Laboratories, Burlingame, CA, USA) before colour development with 3,3′-diaminobenzidine (DAB) (MilliporeSigma, Burlington, MA, USA). SMI312 immunostaining of phosphorylated neurofilament medium and heavy was performed using the M.O.M. kit (Vector Laboratories) following the manufacturer’s instructions, before applying the ABC kit and DAB. Aβ was immunodetected with 6E10 using procedures similar to SMI312, but with the extra initial step of incubation in 88% formic acid. APP was immunodetected with 22C11 similar to SMI312, but with an extra step of antigen retrieval by boiling the samples in a pressure cooker using Tris-ethylenediaminetetraacetic acid, pH 8.0, for 8 min. Antibodies and dilutions were as follows: Iba1 (019-19741, 1:1000; Wako Pure Chemical Industries, Tokyo, Japan), SMI312 (837904, 1:1000; BioLegend, San Diego, CA, USA), 6E10 (803015, 1:1000; BioLegend), and 22C11 (MAB348, 1:4000’ MilliporeSigma). Astrocytes were stained using glial fibrillary acidic protein (GFAP) antibody (GA5-488, 53-9892-80, 1:400; Thermo Fisher Scientific, Waltham, MA, USA). Injured axons were stained using the NeuroSilver Staining Kit (FD NeuroTechnologies, Columbia, MD, USA) following the manufacturer’s instructions, and fibrillary amyloid was stained using 1% thioflavin S (ThioS) (MilliporeSigma), as described previously [68, 76].

Entire coronal sections were imaged with Zeiss Axio Scan.Z1 (Carl Zeiss Microscopy, Thornwood, NY, USA) at 20× magnification, using bright field (Iba1, Silver, SMI312) or fluorescent (ThioS, GFAP) imaging. ROIs included grey matter (prefrontal cortex, parietal cortex, hippocampus [CA1, CA2/3 and dentate gyrus]) and basolateral amygdala and white matter (corpus callosum and optic tract). Image quantification was performed using ImageJ software (National Institutes of Health, Bethesda, MD, USA). For Iba1 staining, images were quantified by thresholding and reporting the number of microglia per area of the optic tract, after filtering background noise of particles less than 27 μm^2^. Microglia with sizes from 27 to 144 μm^2^ were considered non-activated, and those with sizes of 145 μm^2^ or above were considered activated. SMI312-stained images were quantified by thresholding and reporting the number of axonal swellings per area of the optic tract after filtering background noise of particles < 21 μm^2^ or with circularity < 0.2. 6E10 and ThioS images were quantified by thresholding and reporting the percentage area containing signal relative to the total cortical area. Silver-stained images were quantified by thresholding and reporting the percentage area containing signal relative to the white matter area.

### Biochemistry

Biochemical procedures were carried out as reported previously [17]. Briefly, unfixed hemibrains were homogenized in 1.5 ml of ice-cold carbonate buffer (100 mM Na_2_CO_3_, 50 mM NaCl, pH 11.5) containing cOmplete protease inhibitor (Roche Diagnostics, Mannheim, Germany), 1 mM phenylmethanesulfonyl fluoride (PMSF) and PhosSTOP (Roche) in a TissueMite homogenizer (Teledyne Tekmar, Mason, OH, USA) at full speed for 20 s and then sonicated at 20% output for 10 s. After incubating on ice for 10 min, lysates were separated by centrifugation at 16,600 *g* for 45 min at 4 °C. The carbonate-soluble fraction was extracted and neutralized with 1.5 vol of 1 M Tris-HCl, pH 6.8, to achieve a final pH of approximately 7.4. The insoluble fraction was then resuspended in 1.5 ml of 5 M guanidine hydrochloride (GuHCl) in 50 mM Tris-HCl, pH 8.0, at room temperature for 3 h with continuous rotation. All samples were frozen at − 80 °C until use. Protein concentration was determined using a Lowry protein assay (Bio-Rad Laboratories, Hercules, CA, USA).

Human Aβ40 and Aβ42 levels in carbonate and GuHCl fractions from APP/PS1 mice were assayed using commercial enzyme-linked immunosorbent assay (ELISA) kits (KMB3482, KMB3442; Life Technologies, Carlsbad, CA, USA) at dilutions 1:4–1:10 for carbonate-soluble and 1:250–1:1000 for GuHCl-soluble assays. Signals were read using Infinite M200 Pro (Tecan, Männedorf, Switzerland). ELISA data points were interpolated from the relevant standard curve using four-parameter nonlinear regression curve fitting and normalized to total protein concentration. Interleukin-6 (IL-6), IL-1β, and tumour necrosis factor α (TNF-α) levels were assayed using a customized murine V-PLEX Proinflammatory Panel 1 (K152A0H-2; Meso Scale Diagnostics, Rockville, MD, USA) at 1:2 dilution and overnight incubation. Total tau and phosphorylated (Thr231) tau were assayed using the MULTI-SPOT assay (K15121D-2; Meso Scale Diagnostics) at 1:50 dilution. Signals were read on a Sector S600 plate reader (Meso Scale Diagnostics), and concentrations were normalized to total protein concentration where applicable. Plasma NF-L levels were measured using the NF-L Advantage Assay (catalogue no. 102258; Quanterix Corp., Lexington, MA, USA) on the Simoa HD-1 Analyzer (Quanterix Corp.) according to the manufacturer’s protocol. Samples were run in singlicate and manually diluted 4× offboard.

### Soluble Aβ antibody binding assay

An Octet RED system (ForteBio, Fremont, CA, USA) equipped with streptavidin biosensors was used to analyse soluble Aβ binding activity in brain extracts. Briefly, aducanumab, an Aβ-specific antibody that binds to soluble fibril fragments and/or high-molecular-weight oligomers, isotype IgG immunoglobulin controls, and Poly8029, an antibody that binds to the N-terminal 1–16 amino acid residue of Aβ, were biotinylated and loaded on the streptavidin biosensors. After quenching residual streptavidin sites and equilibrating to baseline, antibody-loaded biosensors were dipped in brain extracts (diluted 1:5), and binding was monitored for 20 min. Binding responses were normalized to the amount of antibody loaded in each biosensor.

### Statistics

All animal groupings were blinded during analysis by using surrogate identifying codes. Statistical analyses were performed using IBM SPSS Statistics version 23 software (IBM, Armonk, NY, USA), and graphs were plotted using Prism version 6.07 software (GraphPad Software, La Jolla, CA, USA). Mixed linear models were used to analyse RR, PA, EPM, and BM probe and reverse trials, owing to missing values caused by premature deaths. Fixed factors included genotype (APP/PS1 or WT), injury (TBI or sham) and time (various testing days depending on experiments). Each mouse was considered a subject and accounted for random effects. In BM acquisition, BM reverse and RR, there was an extra fixed factor, trial, because multiple separate observations were made on the same day. BM cognitive score and LRR data were analysed using a repeated measures generalized linear model, with the independent variables of genotype and injury, and time as the repeated measures variables. Analysis of the frequency of using direct or random strategies were performed using logistic regression. For cytokine assays, samples below the detection limit were assigned a value of zero, and data were analysed using Kruskal-Wallis test, with post hoc analyses using the Mann-Whitney *U* test and Bonferroni correction for multiple comparisons. All other histological, biochemical and BM probe data were analysed by univariate analysis of variance, with independent variables including group and time (if applicable). For 6E10 and ThioS histology, analyses were performed on fold change over respective average sham level in each experiment. In all analyses, post hoc tests were performed between pairs of the groups if the group factor or Group × time interaction reached statistical significance (*p* < 0.05). A summary of the *p* values of statistical tests performed for experiments and assays in this study is listed in Additional file 2.

## Results

### CHIMERA rmTBI produces no significant neurological or motor deficits in WT and APP/PS1 mice

We previously reported the acute effects of mild TBI using 2 × 0.5 J CHIMERA impacts to hybrid C57BL/6-C3H WT and APP/PS1 mice [17]. The present study was specifically designed to assess the chronic effects of 2 × 0.5 J CHIMERA impacts 8 months after injury in littermate WT and APP/PS1 animals, both on a F1 C57BL/6-C3H background (Additional file 1A). With an average *N* = 12 animals per group, post-procedure LRR scores did not differ between sham or TBI WT and APP/PS1 groups (injury *p* = 0.358, genotype *p* = 0.848, injury × genotype interaction *p* = 0.784), consistent with our previous observations of very mild injury on the C57BL/6-C3H background (Additional file 1B). Both sham and TBI groups recovered from anaesthesia earlier on the second injury day (time *p* = 0.017). Assessment of NSS scores from baseline to D7 post-TBI also showed no significant injury effect (*p* = 0.173) but revealed a significant genotype difference (*p* < 0.001), suggesting that APP/PS1 mice generally had a worse NSS than WT controls, regardless of injury status (Additional file 1C). Although APP/PS1 mice performed worse on the RR task than WT mice (genotype effect *p* = 0.004), neither injury (*p* = 0.100) nor genotype × injury interaction (*p* = 0.374) was significant (Additional file 1D). These observations of acute behaviour confirm a very subtle injury in this cohort, likely in the subconcussive range. Consistent with observations on several APP transgenic lines [52], overall mortality was greater in APP/PS1 mice than in WT mice (Fisher’s exact test *p* = 0.032) but not significantly affected by injury (Fisher’s exact test *p* = 1.000) (Additional file 1E).

### Extinction of fear memory is chronically impaired in APP/PS1 but not in WT mice after CHIMERA rmTBI

**Fig. 1:**
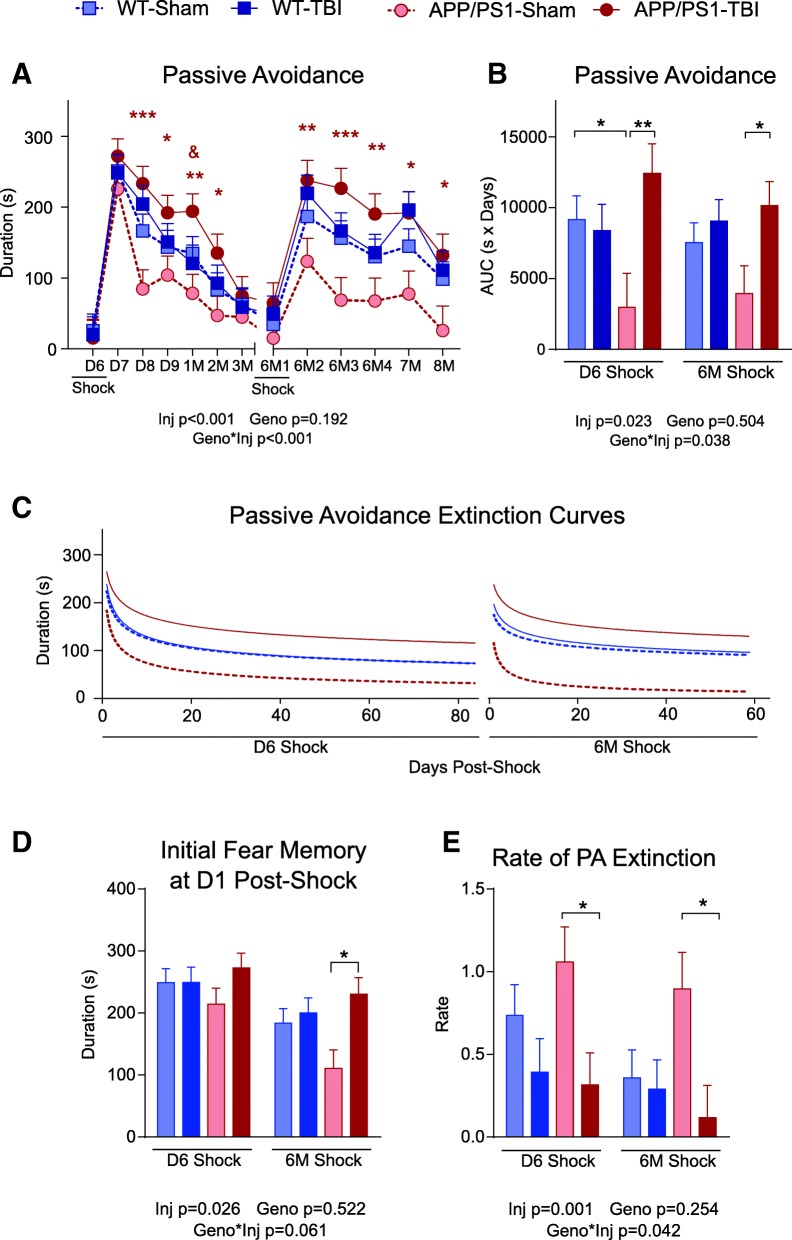
Passive avoidance task performance. **a** On day 6 (D6) post-TBI, mice were placed into the light chamber, and a foot shock was given once they entered into the dark chamber. From D7 to 3 months (3 M) post-TBI, the mice were placed into the light chamber, and the duration of time spent before entering into the dark chamber is reported. No foot shock was given on post-shock days. The experiment was repeated again from 6 M to 8 M post-TBI, where a foot shock was given on the first day of 6 M only. The duration of time spent before the mice entered into the dark chamber is reported. A longer duration indicates stronger fear memory. **b** The cumulative fear response was reported as the AUC from (**a**). **c** The duration in the light chamber on post-shock days was used to fit the Ebbinghaus saving function M = θt^-ψ^ to model the extinction of fear memory, where θ is the initial memory and ψ is the rate of extinction. **d** Initial memory at D1 post-shock was evaluated from (**c**) by inputting *t* = 1. **e** The rate of extinction ψ from (**c**) is reported. Data are expressed as mean ± SE. Omnibus statistical results are provided below each panel. In (**a**), asterisks represent significant post hoc differences between APP/PS1-TBI and APP/PS1-Sham (* *p* < 0.05, ** *p* < 0.01, *** *p* < 0.001). Ampersand represents significant post hoc differences between APP/PS1-TBI and WT-TBI (^&^ *p* < 0.05). In (**b**), (**d**) and (**e**), asterisks represent significant post hoc differences between marked groups (* *p* < 0.05, ** *p* < 0.01, *** *p* < 0.001). *n* = 8–13 per genotype per injury per time point

Several behavioural tests revealed chronic impairments after CHIMERA rmTBI. APP/PS1 mice performed worse than WT controls after rmTBI in some tests, particularly for extinction of fear memory evaluated using the PA task, which was conducted from D6 to 8 M post-TBI across two cycles of foot shock and testing (Fig. 1a). The first foot shock (Shock 1) was delivered on D6 post-TBI and PA performance was followed from D7 to 3 M post-TBI. After a 3-month rest interval to minimize task acclimatization, a second foot shock (Shock 2) was given at 6 M after TBI, and PA performance was followed for 3 consecutive days and then monthly to 8 M. Overall PA performance showed a similar pattern after both foot shocks. The two prominent peaks of PA duration after both shock days demonstrated that each group of animals was able to learn fear during both shock events and that each group showed a gradual reduction in post-shock PA duration over time as the acquired fear memory was extinguished. Omnibus analysis of PA duration revealed significant effects of time (*p* < 0.001), injury (*p* < 0.001) and genotype × injury interaction (*p* < 0.001). Post hoc comparisons revealed that WT-TBI and WT-Sham groups were not different (*p* = 0.225), suggesting that CHIMERA rmTBI did not affect fear learning and extinction in WT mice. However, the APP/PS1-Sham group showed the lowest post-shock duration to enter the dark chamber of the four groups (*p* < 0.001 compared with APP/PS1-TBI and WT mice), consistent with impaired memory in APP/PS1 compared with WT mice. Additionally, after the second shock, the APP/PS1-Sham group had a significantly lower peak than the other groups, confirming impaired learning in older APP/PS1 mice. Strikingly, the APP/PS1-TBI group consistently exhibited the longest duration to enter the dark chamber (*p* < 0.001), demonstrating that very mild TBI can alter fear memory in animals genetically engineered to produce human Aβ.

Several lines of evidence suggest that TBI in APP/PS1 mice prolongs extinction of fear memory. The peak duration to enter the dark chamber on D1 after each shock is similar between APP/PS1-TBI and WT-TBI groups, arguing against intensified fear acquisition after rmTBI. Analysis of the temporal profile of PA duration among the four groups showed that APP/PS1-TBI mice demonstrated the slowest recovery and that it remained significantly elevated for 2 months after each shock. AUC analysis confirmed that, after both Shock 1 and Shock 2, APP/PS1-Sham mice had the lowest cumulative fear response (*p* < 0.0001), whereas the APP/PS1-TBI group had the strongest cumulative fear response (*p* < 0.0001) (Fig. 1b). We also modelled the extinction phases of the PA curves with the Ebbinghaus power function [93, 94] (Fig. 1c) to analyse initial fear memory (Fig. 1d) and its extinction rate (Fig. 1e). This analysis confirmed that all four groups displayed comparable initial fear memory after Shock 1, whereas after Shock 2, APP/PS1-TBI mice had significantly stronger initial fear memory than APP/PS1-Sham (*p* = 0.005), which is likely driven by the poorer acquisition of fear memory in the APP/PS1-Sham group, especially in response to Shock 2 (Fig. 1d). Although the extinction rates of WT-Sham and WT-TBI were not significantly different for either shock (*p* = 0.219 and *p* = 0.786, respectively), APP/PS1-TBI mice had a significantly lower extinction rate than APP/PS1-Sham mice after each shock (*p* = 0.013 and *p* = 0.012, respectively). These results suggest that APP/PS1-TBI had greatest cumulative fear response over 8 M post-injury, which is mainly due to their inability to extinguish fear memory.

### Spatial learning and memory are impaired after CHIMERA rmTBI

**Fig. 2:**
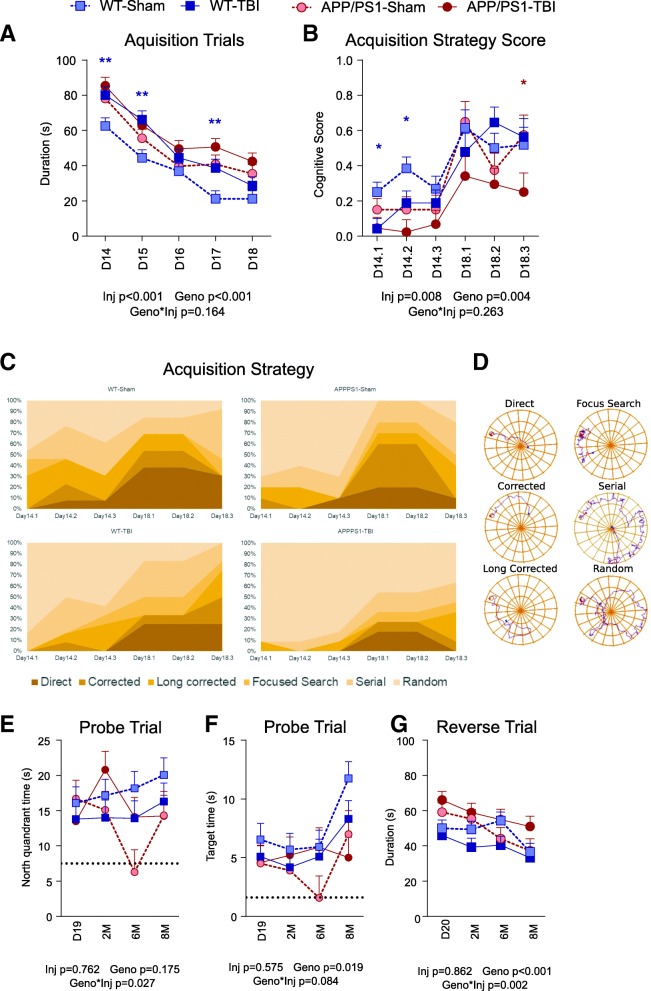
Barnes maze task performance. **a** From D14 to D18 post-TBI, acquisition learning trials were performed, and the time it took to locate and enter into the escape box was reported. The average performance of three trials per day was expressed as mean ± SE. A shorter duration indicates faster spatial learning. **b** The exploration paths of D14 and D18 (the first and last acquisition days, respectively) were analysed and classified into six strategies. The best strategy (direct) was given a score of 1 and the worst (random) a score of 0. The performance during the three trials on each day was plotted as separate data points. A higher score indicates a better exploration strategy. **c** The frequency of employing each strategy was plotted. **d** An example of each exploration strategy is provided. **e** Probe trials were performed on D19, 2 M, 6 M, and 8 M post-TBI, during which the escape box was removed. The time spent inside the north quadrant (the previous escape box location) is plotted. The dotted line represents the expected amount of time that would have been spent by random. A longer time duration indicates better spatial memory. **f** The time spent around the previous escape box location is plotted. The dotted line represents the expected amount of time spent by randomly exploring each possible box location. A longer duration indicates better and more precise spatial memory. **g** Reverse trials were performed on D20, 2 M, 6 M, and 8 M post-TBI, in which the escape box was placed at the opposite location. A shorter duration indicates better spatial unlearning and relearning. Data are expressed as mean ± S.E. Statistical results are provided below each panel. Blue and red asterisks represent significant post hoc differences between WT-Sham and WT-TBI and between APP/PS1-Sham and APP/PS1-TBI, respectively (* *p* < 0.05). *n* = 10–13 per genotype per injury per time point

We used the BM to study spatial learning and memory after CHIMERA rmTBI, where acquisition trials were performed from D14 to D18 post-injury and probe and reverse trials were performed from 1 M to 8 M post-injury. Acquisition trials revealed a significant injury effect (*p* < 0.001) and a significant genotype effect (*p* < 0.001), indicating that APP/PS1 mice learn more slowly than WT mice and that TBI mice learn more slowly than sham mice (Fig. 2a). However, there was no significant genotype × injury interaction (*p* = 0.164), and post hoc analysis showed that both APP/PS1-Sham and APP/PS1-TBI mice took longer to learn than WT-Sham and WT-TBI mice, respectively (*p* < 0.001 and *p* = 0.033). These results suggest that in this small cohort, APP/PS1 genotype and rmTBI had additive rather than synergistic effects on spatial learning.

Further analysis was performed to classify the strategies used by the mice to explore the BM. The exploration paths of the first acquisition day (D14) and fifth acquisition day (D18) were analysed because they were the starting and ending dates of the acquisition trials, which showed the maximum difference between groups. The combined acquisition strategy score, which is a quantification of the exploration strategies, showed a significant injury effect (*p* = 0.008) and a significant genotype effect (*p* = 0.004) (Fig. 2b). These results suggest that APP/PS1-TBI mice, and to a lesser extent WT-TBI mice, have poorer spatial learning and a reduced ability to use an optimal strategy in navigating in the BM. Deeper analysis of search strategy (Fig. 2c) showed that during the first acquisition day (D14), WT-Sham mice used the random approach most frequently (36%) but the direct approach least frequently (5%). By the final acquisition day (D18), the frequency of the random approach decreased to 13%, whereas the direct approach progressively increased to 36%. APP/PS1-Sham mice showed a similar pattern but with more use of the random approach on the first day (67%) and less use of the direct approach (17%) on the final day. The four groups had significantly different search strategies (*p* < 0.001). In WT-TBI mice, the random approach was by far the most dominant search strategy on the first acquisition day (64%), and there was less effective evolution of other search strategies, such that the direct approach was used only 25% of the time on the final day. APP/PS1-TBI showed the most robust impairments, using 88% random strategy on the first acquisition day, and only 12% direct strategy on the final acquisition day. Of all the groups, APP/PS1-TBI animals were the least likely to use the direct approach (eight times less than WT-Sham, *p* = 0.010; five times less than WT-TBI, *p* = 0.053; seven times less than APP/PS1-Sham, *p* = 0.022) and the most likely to use the random approach (seven times greater than WT-Sham, *p* < 0.001; four times greater than WT-TBI, *p* = 0.001; three times greater than APP/PS1-Sham, *p* = 0.002).

Probe trials were used to test long-term spatial memory up to 8 M post-injury. Analysis of time spent in the north quadrant containing the previous target revealed no genotype or injury effects (*p* = 0.175 and 0.762, respectively) but a significant genotype × injury interaction (*p* = 0.027) (Fig. 2d). Post hoc analyses showed that WT-Sham mice performed significantly better than APP/PS1-Sham mice (*p* = 0.013) and tended to perform better than WT-TBI mice (*p* = 0.081). Spatial memory in APP/PS1 mice was not further reduced by rmTBI, because APP/PS1-TBI did not differ from APP/PS1-Sham (*p* = 0.204). By 8 M post-injury, spatial memory performance appeared to stabilize such that the WT-Sham group tended to perform better than the WT-TBI, APP-Sham and APP-TBI groups. Analysis of time spent near the previous target location revealed a significant genotype effect (*p* = 0.019) but no injury effect (*p* = 0.575) or genotype × injury interaction (*p* = 0.084) (Fig. 2e). By 8 M post-injury, time spent in the target location tended to be greatest in WT-Sham group, followed by WT-TBI, APP/PS1-Sham and APP/PS1-TBI groups. Finally, reverse trials were used to investigate cognitive flexibility up to 8 M post-injury and revealed a significant genotype effect (*p* < 0.001) and a significant genotype × injury interaction (*p* = 0.002) but no overall injury effect (*p* = 0.862) (Fig. 2f). Post hoc analyses showed that sham mice of both genotypes performed similarly (*p* = 0.724), whereas APP/PS1-TBI mice performed worse than WT-TBI mice (*p* < 0.001). By 8 M post-injury, the APP/PS1-TBI group tended to show the worst performance of all groups in the reverse trial.

### rmTBI reduces anxiety and increases risk-taking behaviour

**Fig. 3:**
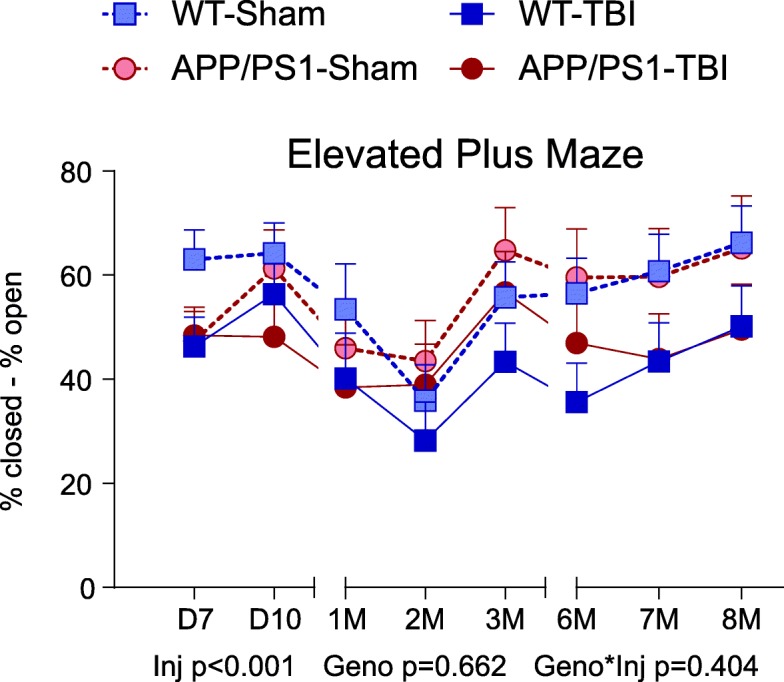
Elevated plus maze task performance. From D7 to 8 M post-TBI, mice were tested in the EPM. The difference between the time spent in the closed arms and the open arms are plotted and expressed as mean ± SE. A greater value indicates more time spent in the closed arms and less time in the open arms, suggesting greater anxiety. A smaller value indicates that the mice spent relatively more time in the open arms and less time in the closed arms, suggesting greater risk-taking behaviour. *n* = 6–13 per genotype per injury per time point

The EPM was used to assess chronic anxiety and risk-taking behaviour by quantifying the percentage of time spent in the closed vs. open arms of the maze D7 up to 8 M post-injury. Omnibus analysis revealed a significant injury effect (*p* < 0.001) but no significant genotype effect (*p* = 0.662) or genotype × injury interaction (*p* = 0.404) (Fig. 3). These data show that that injured mice of both genotypes demonstrated a preference for the open arms over the closed arms of the EPM. Particularly noteworthy is that the distinction between sham and TBI groups became more pronounced over time and stabilized at 6 M and 8 M months post-injury. When EPM data were analysed separately using either time spent in closed arms or time spent in open arms, the same observations were found (injury effect *p* < 0.001 and *p* = 0.001, respectively) (Additional file 1: Figure S1).

### CHIMERA rmTBI induces chronic white matter microgliosis, axonal injury and astrogliosis independent of genotype in the optic tract but not the corpus callosum

**Fig. 4:**
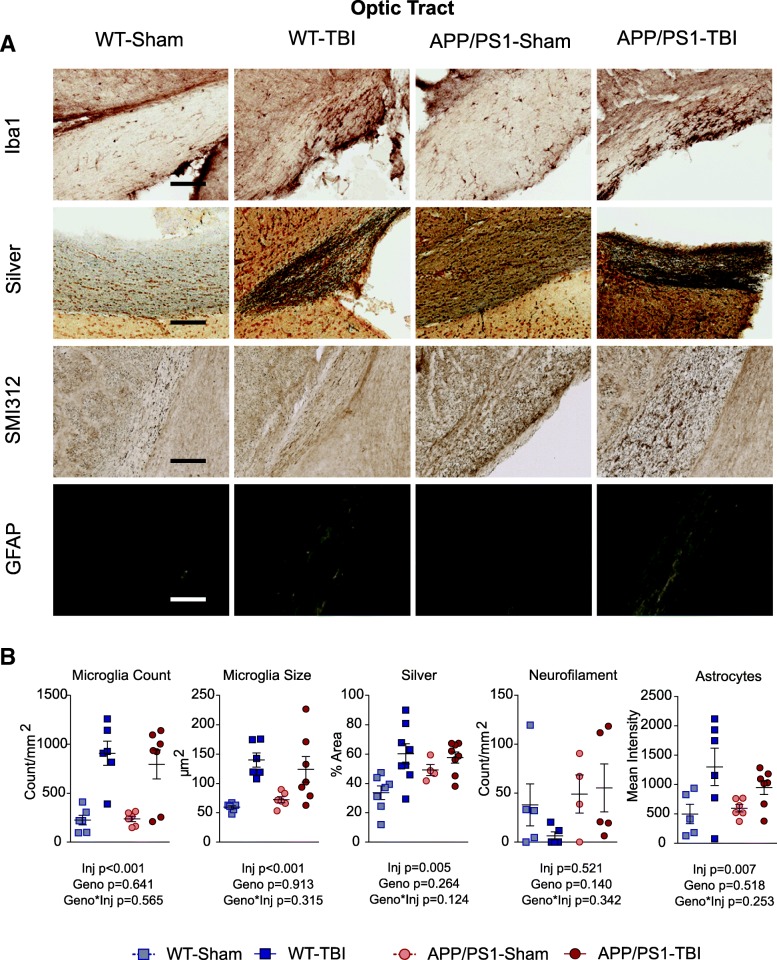
White matter pathology in the optic tract. **a** At 8 M post-TBI, histopathological analyses were performed on the optic tract, using Iba1 (microglia), NeuroSilver (axonal injury), SMI312 (neurofilament), and GFAP (astrocytes). Representative images for each stain are shown. Scale bar represents 200 μm. **b** Quantification of (**a**) was plotted by reporting the density and size of microglia, the stain area of NeuroSilver, the density of neurofilament-positive axonal bulbs, and GFAP immunofluorescence intensity of astrocytes. Data are plotted as mean ± SE with an overlaid scatterplot of individual animals

**Fig. 5:**
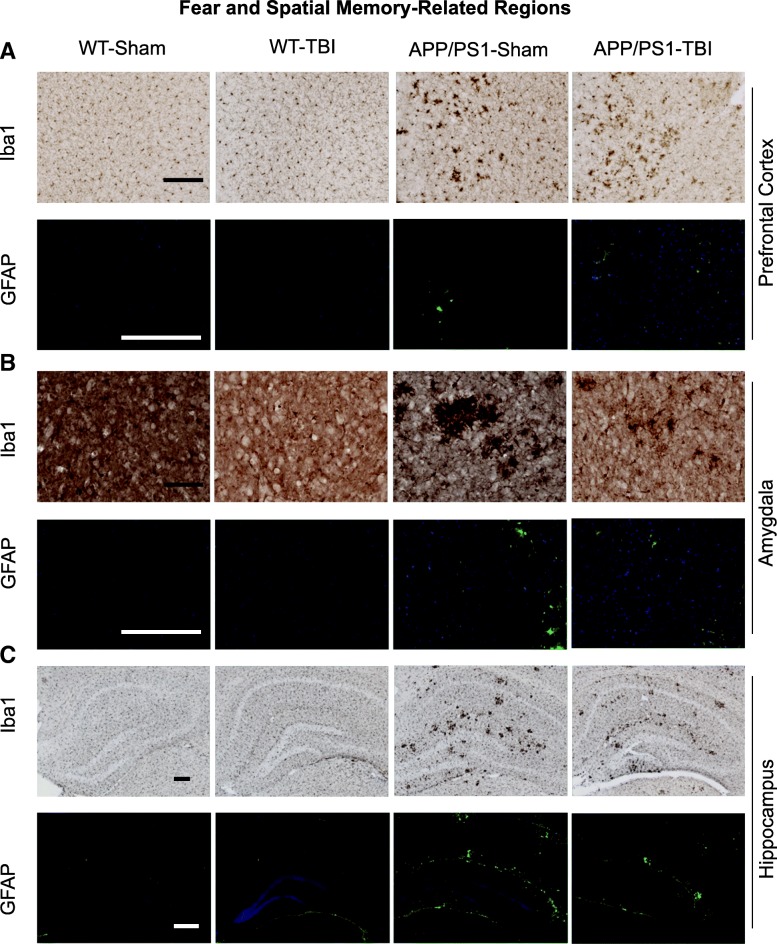
Grey matter pathology at fear and spatial memory-related regions. At 8 M post-TBI, histopathological analyses were performed for (**a**) prefrontal cortex, (**b**) amygdala, and (**c**) hippocampus, using Iba1 and GFAP. Scale bar represents 20 μm for amygdala Iba1 and 200 μm in all other images

We have previously shown that CHIMERA is a reliable model of diffuse axonal injury at acute time points up to D14 post-injury in multiple white matter regions [66, 68]. Here we examined the optic tract and corpus callosum as representative white matter regions at 8 M post-injury. In the optic tract (Figs. 4 and 5), Iba-1 IHC revealed that CHIMERA rmTBI led to significantly increased microglial density (*p* < 0.001) and size (*p* = 0.005) in both WT and APP/PS1 mice, without significant effects of genotype or of genotype × injury interaction. NeuroSilver staining also revealed a significant effect of injury (*p* = 0.005) but not genotype (*p* = 0.264) or genotype × injury interaction (*p* = 0.124). We previously showed that neurofilament bulbs were formed acutely after CHIMERA rmTBI in young (aged 6 months) but not old (aged 13 months) WT or APP/PS1 mice after rmTBI, and such bulbs were present only at D2 but not D7 post-injury [17]. Consistent with our previous observations, we did not observe a significant increase in rmTBI-induced SM132 staining 8M after injury in the 14-month-old cohort examined here, but rather observed a trend toward more SM132 reactivity with higher variability in APP/PS1 animals. Immunostaining for APP using 22C11 showed no differences in the optic tract across the four groups (not shown). Finally, GFAP staining revealed significantly increased optic tract astrogliosis in both WT-TBI and APP/PS1-TBI (injury effect *p* = 0.013), but no significant genotype effect (*p* = 0.518) or interaction (*p* = 0.253). Compared with the optic tract, the corpus callosum appeared more resilient to injury or better able to recover from injury, because no significant differences were observed among the four groups for 22C11 (not shown), microglia count, microglia size, or NeuroSilver and SM132 staining 8 months post-injury (Additional file 3). In the corpus callosum, APP/PS1 mice had significantly increased GFAP staining compared with WT controls (*p* < 0.001), but this was unaffected by rmTBI (*p* = 0.661) (Additional file 3).

### rmTBI alters microgliosis in APP/PS1 amygdala and hippocampus

**Fig. 6:**
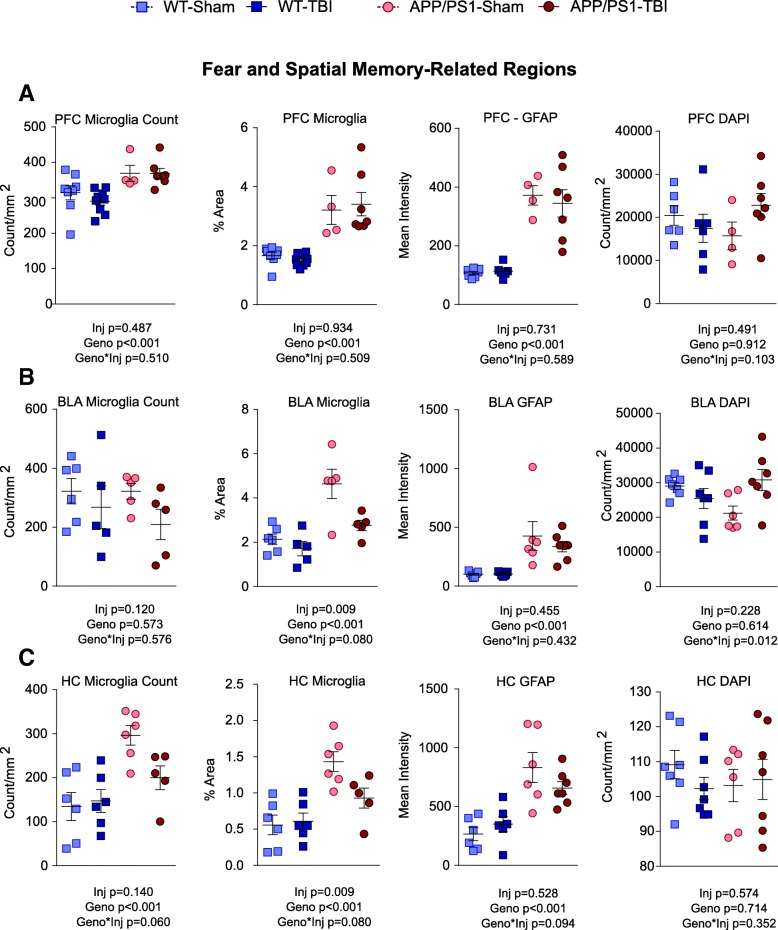
Quantification of grey matter pathology at fear and spatial memory-related regions. On the basis of images in Fig. 5, the total density of microglia, the stained area of all microglia, the mean intensity of GFAP fluorescence, and the count of total nucleus were plotted for (**a**) prefrontal cortex, (**b**) amygdala, and (**c**) hippocampus. Data are plotted as mean ± SE with an overlaid scatterplot of individual animals

**Fig. 7:**
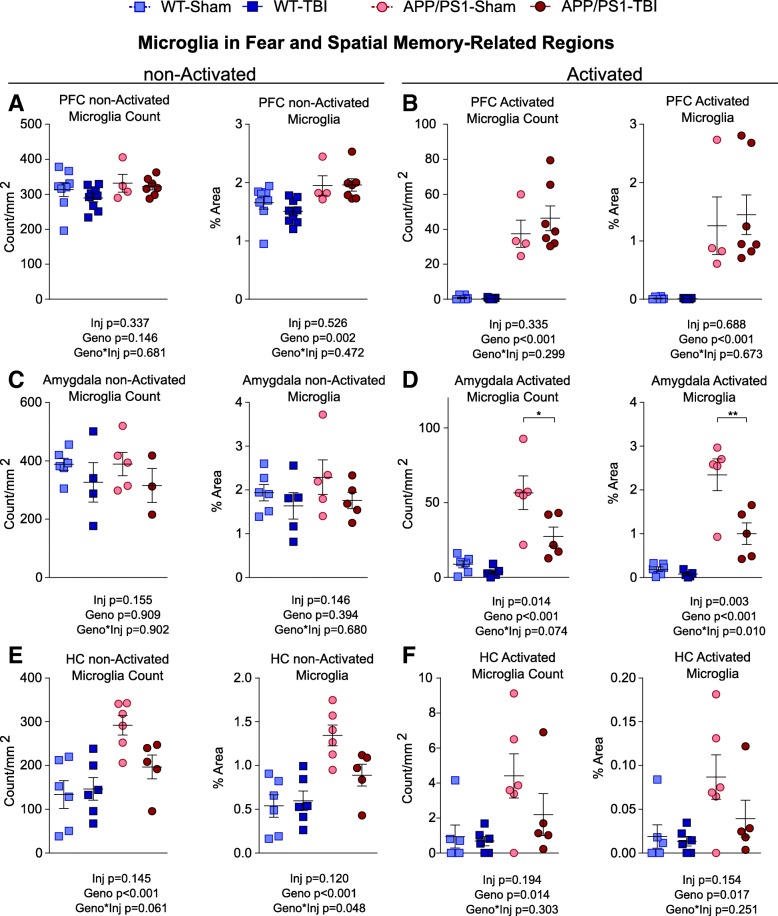
Quantification of non-activated microglia at fear and spatial memory-related regions. Because microglia in grey matter regions appear as either a non-activated ramified morphology with a size of less than 145 μm^2^ or an activated morphology with a size greater than 145 μm^2^, the 145-μm^2^ size was used as a surrogate cut-off for non-activated and activated microglia. The density and stain area for each type of microglia are plotted for (**a**, **b**) prefrontal cortex, (**c**, **d**) amygdala, and (**e**, **f**) hippocampus. Data are shown as mean ± SE with an overlaid scatterplot of individual animals. Asterisks indicate significant post hoc difference between APP/PS1-TBI and APP/PS1-Sham (* *p* < 0.05; ** *p* < 0.01)

Although grey matter changes have not been observed with mild CHIMERA injuries at acute time points [17], the robust chronic behavioural changes observed in this study prompted us to examine several grey matter regions involved in learning and memory, including the prefrontal cortex, amygdala and hippocampus (Figs. 5, 6 and 7). Using Iba1 IHC, we found that APP/PS1 mice displayed greater microgliosis than WT littermates. APP/PS1 mice had significantly greater microglia stain area than WT (*p* < 0.001) in all three regions examined (Fig. 6), and APP/PS1 mice had significantly greater total microglia number than WT (*p* < 0.001) in the prefrontal cortex and hippocampus.

For injury effects, in the prefrontal cortex, CHIMERA rmTBI did not significantly alter microglia count or stain area in WT or APP/PS1 mice (injury effect *p* = 0.487 and *p* = 0.934, respectively; injury × genotype interaction *p* = 0.510 and *p* = 0.509, respectively) (Fig. 6a). In the amygdala, rmTBI had a significant injury effect (*p* = 0.009) in microglia stain area and a trend towards significant injury × genotype interaction (*p* = 0.080) in total microglia stain area, suggesting that rmTBI led to a chronic reduction in total microglia stain area specifically in APP/PS1 mice.

Because most microglia in the grey matter of WT mice had a non-activated ramified morphology with an area < 145 μm^2^ in size, we decided to separate microglia based on size (non-activated, < 145 μm^2^; activated, ≥ 145 μm^2^) in further analyses (Fig. 7). We observed that, again in the amygdala of APP/PS1 mice, rmTBI significantly reduced the stain area of activated microglia (injury × genotype interaction *p* = 0.010) (Fig. 7d). There was also a trend towards significant reduction of activated microglia number in APP/PS1 (injury × genotype interaction *p* = 0.074). However, rmTBI did not change the stain area and count of non-activated microglia in the amygdala (Fig. 7c). In the hippocampus of APP/PS1, rmTBI had a trend towards decreasing the number and area of activated microglia, but it did not reach significance (Fig. 7f). Interestingly, in the hippocampus of APP/PS1 mice, rmTBI significantly reduced the area of non-activated microglia (injury × genotype interaction *p* = 0.048) and had a trend towards significantly reducing the number of non-activated microglia (injury × genotype interaction *p* = 0.061) (Fig. 7e).

For GFAP, staining was significantly greater in APP/PS1 compared with WT animals in all regions (*p* < 0.001) but not affected by injury in either WT or APP/PS1 mice (Fig. 6). Despite these immunohistological changes suggestive of altered neuroinflammation, no significant effects in IL-1β, IL-6 and TNF-α protein levels in carbonate-soluble brain homogenates were observed in TBI and sham groups of either genotype (Additional file 4A).

4′,6-diamidino-2-phenylindole (DAPI) stain revealed an increase in the number of nuclei in the amygdala of APP/PS1-TBI compared with other groups (genotype × injury interaction *p* = 0.012), despite no significant increase in the number of astrocytes or microglia in the same region. Interestingly, other mTBI models have shown that TBI-induced conditioned fear can be associated with an increased neuronal number in the amygdala [62], though in our present study we have not identified the specific cell type or neuronal population that results in the current increase of DAPI signals.

### rmTBI does not alter long-term tau, Aβ or amyloid levels, but increases aducanumab binding activity

**Fig. 8:**
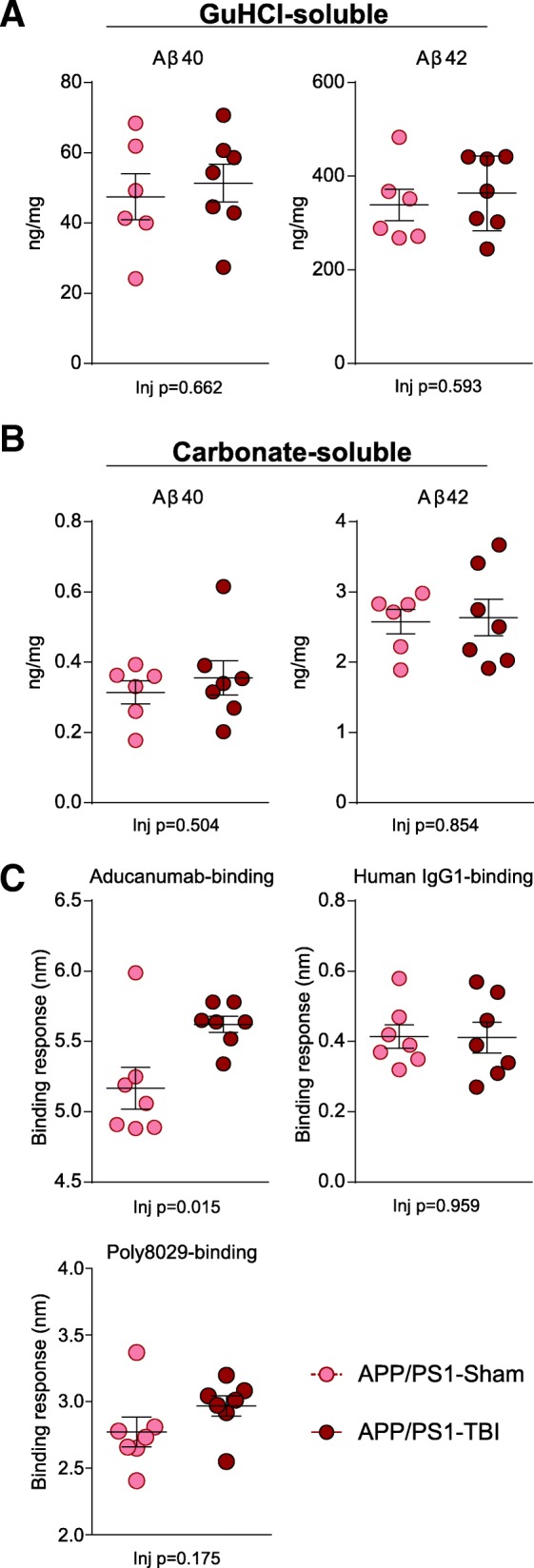
Aβ analyses. Brain homogenates from mice harvested at 8 M post-TBI were serially extracted in carbonate and then guanidine HCl (GuHCl) buffer. **a** The concentration of Aβ40 and Aβ42 in the GuHCl-soluble fraction is plotted. **b** The concentration of Aβ40 and Aβ42 in the carbonate-soluble fraction is plotted. **c** Using an Octet RED system with streptavidin biosensors, the levels of aducanumab-binding high-molecular-weight oligomeric and soluble fibril fragment forms of Aβ and the total level of Poly8029-binding Aβ were assayed. Human IgG1 served as an isotype control for aducanumab. Data are plotted as mean ± SE with an overlaid scatterplot of individual animals

Meso Scale Diagnostics ELISA analyses were performed on the carbonate-soluble fraction to quantify murine total tau and p-Thr231 tau levels in each group. Although we observed a significant genotype effect (*p* = 0.024 and *p* < 0.001, respectively) because both analytes were lower in APP/PS1 than in WT mice, rmTBI did not affect total tau, p-tau, or the p-tau/total tau ratio (Additional file 4B). Histological analysis of Aβ and fibrillary amyloid burden was performed using 6E10 (Additional file 5) and ThioS staining (Additional file 6), respectively, in the parietal cortex, prefrontal cortex, basolateral amygdala, hippocampus and corpus callosum of APP/PS1 mice 8 M after sham or rmTBI. No significant injury effects were observed in any region, consistent with our previous observation of only transient and subtle changes in plaque morphology at acute time points after CHIMERA rmTBI [17]. Western blotting showed that APP/PS1 mice had significantly higher levels of sAPP and APP-CTF (*p* < 0.001) than WT mice, but no injury effects were observed (*p* = 0.740 and *p* = 0.399) (Additional file 7). Consistent with no injury effects on APP levels or C-terminal fragment (CTF) production, human Aβ40 and Aβ42 ELISAs performed on brain homogenates from APP/PS1 mice also revealed no significant injury effect in carbonate-soluble (*p* = 0.504 and *p* = 0.854, respectively) or guanidine HCl-soluble (*p* = 0.662 and *p* = 0.593, respectively) fractions (Fig. 8a, b) compared with sham controls. However, because Aβ levels do not necessarily reflect Aβ functions, we also assayed Aβ binding activity in brain extracts of APP/PS1-Sham and APP/PS1-TBI mice using an Octet RED system loaded with various antibodies, including aducanumab or isotype IgG control biosensors. Intriguingly, this assay revealed significantly greater Aβ binding activity towards aducanumab in the APP/PS1-TBI group (*p* = 0.015), suggesting a greater level of high–molecular-weight Aβ oligomers and/or soluble fibril fragments (Fig. 8c).

## Discussion

This study was designed to assess the chronic behavioural and neuropathological effects of two very mild TBIs delivered to WT and APP/PS1 animals, thereby allowing us to evaluate the influence of human Aβ and amyloid on chronic outcomes in animals otherwise matched for genetic background. The first major finding of our study is that even two very mild injuries (i.e., those that do not result in very notable acute behavioural deficits) can lead to long-lasting changes in behaviour, particularly in the domain of cognitive flexibility. For example, TBI mice, particularly from the APP/PS1 genetic background, had reduced probe trial memory and deficits in reverse trial relearning in the BM, demonstrating that both rmTBI and Aβ affect cognitive flexibility in spatial functions and memory. Fear memory as measured by PA was not affected in WT mice but was profoundly altered in APP/PS1 animals, in which fear memory extinction after TBI was greatly diminished. A classical test of anxiety using the EPM showed that TBI reduced anxiety and increased risk-taking exploration of the open arms in both genotypes. Together, these results suggest that many behaviours can be chronically affected after CHIMERA rmTBI and that some of these are influenced by the presence of human Aβ.

However, the second major finding of our study is that we do not yet know the neuropathological correlates of these chronic behavioural changes, because there is no clear association between Aβ or the white and grey matter changes investigated here with altered behaviour. Specifically, CHIMERA is well established to produce acute diffuse axonal injury in both APP/PS1 and WT mice, and here we show that the optic tract exhibits sustained microgliosis, astrogliosis and axonal damage 8 months after injury. Because sustained microgliosis, astrogliosis and axonal damage were not present in the corpus callosum, our results suggest that different white matter regions may have distinct patterns of recovery from or resilience to rmTBI. We also observed inflammatory changes in several grey matter regions associated with learning and memory, including the prefrontal cortex, basolateral amygdala and hippocampus, but these changes cannot fully explain the altered behaviours we observed. Finally, although rmTBI did not change the overall levels of soluble or insoluble Aβ levels and did not affect Aβ deposition in diffuse or fibrillar deposits, it did lead to a chronic elevation of Aβ binding activity towards aducanumab, suggesting increased formation of high-molecular-weight Aβ oligomers and/or soluble fibril fragments. Whether this shift in Aβ oligomeric/soluble fibril fragment species may underlie the chronic behavioural differences between WT and APP/PS1 mice after rmTBI remains to be determined.

Abnormal fear extinction is a central feature of PTSD [96], which is clinically characterized by involuntary and recurrent fear and altered mood and cognition [85]. Studies in military personnel have revealed an important and complex relationship between TBI and PTSD. Both TBI and PTSD are common in military veterans; TBI is a risk factor for PTSD, and pre-existing PTSD may also affect TBI outcomes [34]. The lifetime prevalence of PTSD in the United States is 6.8% [49], and its incidence after civilian TBI is estimated to range from 14% to 56% [11]. In Iraq and Afghanistan veterans, comorbidity of PTSD and TBI is high and associated with loss of consciousness after TBI [34]. Overall, rates of PTSD after TBI in veterans ranges from 9.1% to 43.9% [34], and mild TBI is associated with a 2.37-fold increase in PTSD prevalence [84].

Although PTSD in humans is a complicated disorder, deficits in fear learning and extinguishing can be modelled in animals [92]. In this study, we used PA, one of the most widely studied forms of fear learning, to probe for deficits in fear behaviour after TBI. The neurocircuitry involved in fear learning in one-trial inhibitory avoidance tasks such as PA has been widely studied. The acquisition, consolidation and extinction of fear memory critically depend on brain regions including the hippocampus, amygdala, and prefrontal cortex, with additional contributions from structures including the entorhinal cortex, perirhinal cortex, and posterior parietal cortex (reviewed in [39]). Processing of the PA task and association of conditioned stimulus (entering the dark chamber) and unconditioned stimulus (foot shock) takes place mainly through the trisynaptic circuit of the hippocampus (dentate gyrus > CA3 > CA1), whereas the emotional component involved in PA consolidation takes place in the basolateral amygdala. The processing of PA is then relayed to the central amygdala, which is the output nucleus of the amygdala and is responsible for the fear response. The retrieval and extinction of conditioned fear, on the other hand, require the function of prelimbic and infralimbic ventromedial prefrontal cortex, respectively. Specifically, during extinction of conditioned fear, the infralimbic ventromedial prefrontal cortex stimulates intercalated cells in the amygdala, which in turn leads to increased inhibitory output from central amygdala. Because the ventromedial prefrontal cortex lies within the expected location of contrecoup injury induced by CHIMERA, it may be vulnerable to mechanical damage during rmTBI. On the other end of the unlearning/relearning spectrum, reconsolidation of fear learning depends heavily on the hippocampus [39].

Our observation that TBI in APP/PS1 but not WT mice chronically enhances the fear response for an extended period of time suggests TBI may exacerbate fear consolidation and extinction in animals predisposed to Alzheimer’s amyloidosis. Our finding resonates with clinical observations. For example, one study followed 181,093 war veterans (53,155 developing PTSD) for a median of 7 years [95] and found that veterans with PTSD were more than twice as likely to develop dementia (OR for dementia in general, 2.31; OR for AD, 1.71; OR for FTD, 2.19) as those without PTSD. Further, a recent PET study in Vietnam War veterans examined the spatial distribution of amyloid in control subjects, subjects with TBI, PTSD or subjects with both TBI and PTSD. All three clinical groups showed increased uptake in the rank order PTSD > both > TBI > control with a parallel rank order for cognitive function. Intriguingly, the groups showed distinct regional differences, as the standardized uptake value ratio increase was widespread in cortical regions of subjects with PTSD, in white matter of subjects with TBI-PTSD and in cerebellum and precuneus of subjects with TBI compared with control subjects [63]. Our experimental data support the idea that there is an association between the development of PTSD and presence of human Aβ. The observation that aducanumab binding activity, indicative of high-molecular-weight Aβ oligomers and/or soluble fibril fragments, but not overall levels or deposition patterns, is chronically altered after rmTBI raises the hypothesis that some form of soluble or oligomeric Aβ may interfere with the neurocircuitry of fear memory and extinction independent of classical measures of Aβ neuropathology.

CHIMERA was developed in 2014 as a reliable non-surgical model of diffuse traumatic axonal injury [66–68]. A recent study reported that CHIMERA repetitive mTBI in C57BL/6 mice induces long-term memory deficits with astroglial and microglial changes up to 6 months post-injury [15]. Here, we demonstrate that behavioural and neuropathological changes after CHIMERA can be observed for at least 8 months after injury. Our behavioural results are strongly supported by those of Come et al., who used fear conditioning to assess PTSD-like behaviours in C57BL/6 mice after open-head controlled cortical impact [19]. They observed that TBI increased the frequency of spontaneous resurgence of conditioned fear when tested for fear extinction memory recall even after the animals had acquired and extinguished conditioned fear 6 weeks earlier than the re-test. Similar to our observations, they also reported increased risk-taking behaviour and cognitive deficits, cognitive inflexibility, and reduced processing speed. Their neuropathological analyses revealed morphological changes in the amygdala 3 months post-injury; decreased myelin basic protein density in the primary lesion site as well as hippocampus, thalamus and amygdala; and decreased brain-derived neurotrophic factor (BDNF) mRNA in the prefrontal cortex.

Although less studied than acute outcomes, TBI leads to several chronic changes in animal models that were recently summarized by Osier [70]. After open-head injury in rodents, reported chronic changes from 1 month to 1 year post-injury include shrinkage of grey matter and neuronal loss (e.g., cortex and hippocampus) [8, 10, 21, 47, 87], enlargement of ventricles [8, 21], shrinkage of white matter, and axonal injury and axonal swelling (e.g., corpus callosum and external capsule) [7, 10, 27, 74, 79, 88]. Chronic behavioural deficits include impairment in motor [36, 54], spatial learning [3, 55, 82, 83] and spatial memory [21, 83]. Prolonged microglial responses in grey matter and white matter have also been reported [1, 9]. In closed-head injury models including CHIMERA, reported chronic behavioural deficits include motor [53], spatial memory [4, 15, 57], and depression and anxiety-like behaviour [32, 72]. Much remains to be learned about how chronic TBI outcomes differ across the wide variety of TBI models (i.e., open head, closed head, blast) and as a function of repetitive injury.

Our study has several important strengths, including the use of CHIMERA as a biomechanically relevant closed-head TBI model and a cohort of F1 WT and APP/PS1 littermates, thereby controlling for genetic background and enabling behaviours that are modified by Aβ to be identified. Specifically, fear memory extinction and spatial memory search strategy are behaviours that are modified by the presence of Aβ, whereas general anxiety/risk-taking behaviour and motor performance are not. Our study also has several limitations, including only endpoint neuropathology in a relatively small cohort, concluding with no clear explanation of chronic behaviours from the neuropathological changes we investigated.

Future studies will therefore be required to define the mechanisms that alter long-term behaviour after TBI and understand how these may or may not relate to neuropathology. Observation of elevated Aβ oligomer/soluble fibril binding activity after TBI in APP/PS1 mice raises some new hypotheses to test. For example, it is known that APP-overexpressing mice are prone to seizures as well as non-convulsive epileptiform activity [6]. PTSD is also associated with psychogenic non-epileptic seizures in veterans [81]. Because seizure activity is associated with altered GABAergic signalling [2], APP/PS1 mice develop age-dependent changes in synaptic dysfunction and altered GABAergic neurotransmission [71], and the A5-GABAα receptor has been identified in mice to modify fear extinction [28], it is possible that rmTBI may affect how Aβ interacts with GABAergic neurotransmission. Another possibility is sleep disruption, which occurs both in AD [86] and after TBI [64] and may influence both neuroinflammation and behaviour [22]. Importantly, sleep quality and emotional regulation predict anxiety in veterans with PTSD [56]. Other factors associated with fear extinction and PTSD, including BDNF [25] and calcium calmodulin kinase II [50], should also be explored. Although much remains to be learned, our study provides proof-of-concept data that the CHIMERA model of TBI will be useful to understand how mild TBI induces chronic behavioural and neuropathological changes, which may have distinct aetiologies.

## Conclusions

CHIMERA is a useful model to investigate the chronic consequences of rmTBI, including behavioural abnormalities consistent with features of PTSD and inflammation of both white and grey matter. The presence of human Aβ greatly modifies extinction of fear memory after rmTBI.

## Additional files

Additional file 1: Study design. **a** Schematic diagram of the study design. Two mild TBIs at 0.5 J were induced at 5 to 6 months of age in APP/PS1 mice and WT littermates. Sham procedures were performed as controls. Mice were followed for 8 M post-injury and longitudinally assessed with various behavioural tasks. **b** LRR duration after TBI or sham injuries is shown. **c** The NSS of the animals at pre-injury and 1 h post-injury, as well as up to 7 days post-injury, is reported. A higher score indicates greater neurological deficits. **d** The percentage of mice that survived the entire 8 M post-injury period is reported. **e** EPM performance is plotted by reporting the time spent in open arms. A higher value suggests stronger risk-taking behaviour. **f** EPM plotted by reporting the time spent in closed arms. A higher value suggests greater anxiety-like behaviour. **g** RR performance of animals. A longer fall latency indicates better motor coordination. In (**d**), data are plotted as percentage of the whole. In all others, data are plotted as mean ± SE. (PDF 176 kb)

Additional file 2: Summary of *p* values. This table summarizes the *p* values of the experiments and assays reported in this study. (DOCX 23 kb)

Additional file 3: White matter pathology in the corpus callosum. **a** At 8 M post-TBI, histopathological analyses were performed on the corpus callosum at the level of the dorsal hippocampus, using Iba1 (microglia), NeuroSilver (axonal injury), SMI312A (neurofilament) and GFAP (astrocytes). Representative images for each stain are shown. (**b**) Quantification of (**a**) is plotted by reporting the density and size of microglia, the stain area of NeuroSilver, the density of neurofilament-positive axonal bulbs, and the GFAP immunofluorescence intensity of astrocytes. Scale bar represents 200 μm. Data are plotted as mean ± SE. (PDF 1515 kb)

Additional file 4: Brain cytokines and tau and plasma neurofilament-light. The carbonate-soluble fraction of brain homogenates were assayed for (**a**) inflammatory cytokines including IL-1β, IL-6, and TNF-α and (**b**) total tau, p-tau at Thr231, and the ratio of p-tau to total tau. **c** Plasma levels of neurofilament-light at 8 M post-injury is also reported. In (**a**), data are plotted as median ± interquartile range. In (**b**) and (**c**), data are plotted as mean ± SE. (PDF 50 kb)

Additional file 5: Aβ deposits in grey and white matter. **a** 6E10 immunostaining for diffuse Aβ deposits was performed for the parietal cortex, fear and spatial memory-related areas, and the corpus callosum. The optic tract was immunonegative for 6E10 staining and thus not shown. **b** Quantification of (**a**) showing the percentage area stained by 6E10. Scale bar represents 100 μm. Data are plotted as mean ± SE. (PDF 1133 kb)

Additional file 6: Amyloid deposits in grey matter. **a** ThioS was used to stain fibrillary amyloid at the parietal cortex and in fear and spatial memory-related areas. **b** Quantification of (**a**) showing the percentage area stained by ThioS. Scale bar represents 100 μm. Data are plotted as mean ± SE. (PDF 151 kb)

Additional file 7: Western blot analysis of Aβ metabolism. **a** Protein levels of soluble APP, APP-C-terminal fragment and GAPDH in carbonate-soluble brain homogenates were analysed by Western blotting. **b** Quantification of (**a**) by densitometry. Data are plotted as mean ± SE. (PDF 44 kb)

## Abbreviations

Aβ: Amyloid-β
AD: Alzheimer disease
BDNF: Brain-derived neurotrophic factor
BM: Barnes maze
CHIMERA: Closed-Head Impact Model of Engineered Rotational Acceleration
CTE: Chronic traumatic encephalopathy
DAB: Diaminobenzidine
ELISA: Enzyme-linked immunosorbent assay
EPM: Elevated plus maze
GCS: Glasgow Coma Scale
GuHCl: Guanidine hydrochloride
IL: Interleukin
LRR: Loss of righting reflex
mTBI: Mild traumatic brain injury
NSS: Neurological Severity Score
PA: Passive avoidance
PMSF: Phenylmethanesulfonyl fluoride
p-tau: Phosphorylated tau
PTSD: Post-traumatic stress disorder
rmTBI: Repetitive mild traumatic brain injury
RR: Rotarod
TBI: Traumatic brain injury
ThioS: Thioflavin S
TNF-α: Tumour necrosis factor α
