# Supplementary material for: CHIMERA repetitive mild traumatic brain injury induces chronic behavioural and neuropathological phenotypes in wild-type and APP/PS1 mice
Source: Alzheimers Res Ther. 2019 Jan 12;11:6. doi: 10.1186/s13195-018-0461-0 (PMC6330571; doi:10.1186/s13195-018-0461-0)
Supplement: Supplementary file 1 — Study design. a Schematic diagram of the study design. Two mild TBIs at 0.5 J were induced at 5 to 6 months of age in APP/PS1 mice and WT littermates. Sham procedures were performed as controls. Mice were followed for 8 M post-injury and longitudinally assessed with various behavioural tasks. b LRR duration after TBI or sham injuries is shown. c The NSS of the animals at pre-injury and 1 h post-injury, as well as up to 7 days post-injury, is reported. A higher score indicates greater neurological deficits. d The percentage of mice that survived the entire 8 M post-injury period is reported. e EPM performance is plotted by reporting the time spent in open arms. A higher value suggests stronger risk-taking behaviour. f EPM plotted by reporting the time spent in closed arms. A higher value suggests greater anxiety-like behaviour. g RR performance of animals. A longer fall latency indicates better motor coordination. In (d), data are plotted as percentage of the whole. In all others, data are plotted as mean ± SE. (PDF 176 kb) [file 13195_2018_461_MOESM1_ESM.pdf]

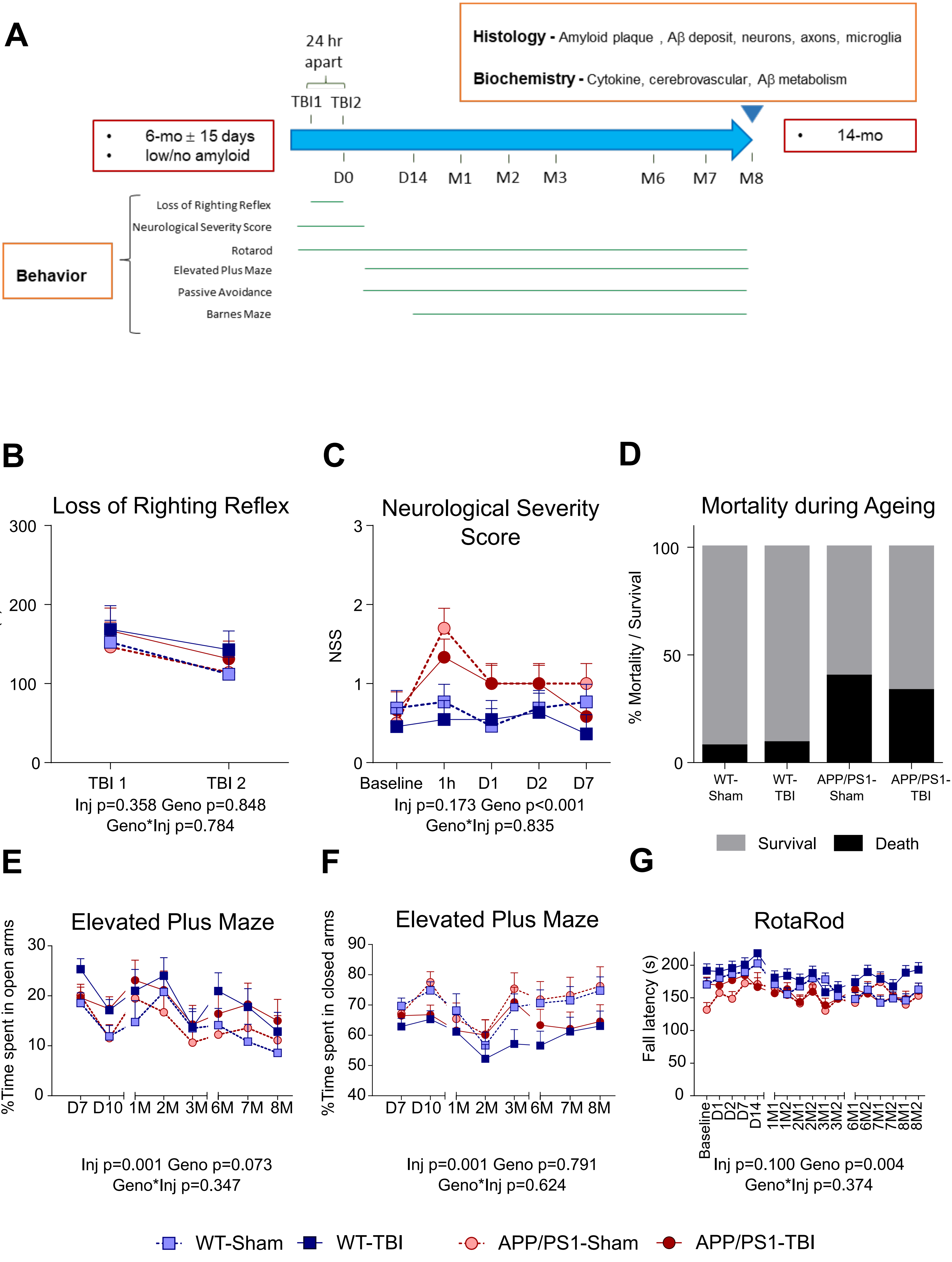

A
24 hr apart
TBI1 TBI2
Histology - Amyloid plaque , Aβ deposit, neurons, axons, microglia
Biochemistry - Cytokine, cerebrovascular, Aβ metabolism
6-mo ± 15 days
low/no amyloid
14-mo
D0 D14 M1 M2 M3 M6 M7 M8
Behavior
Loss of Righting Reflex
Neurological Severity Score
Rotarod
Elevated Plus Maze
Passive Avoidance
Barnes Maze
B
Loss of Righting Reflex
Duration (s)
TBI 1
TBI 2
Inj p=0.358 Geno p=0.848
Geno*Inj p=0.784
C
Neurological Severity Score
NSS
Baseline 1h D1 D2 D7
Inj p=0.173 Geno p<0.001
Geno*Inj p=0.835
D
Mortality during Ageing
% Mortality / Survival
WT-Sham
WT-TBI
APP/PS1-Sham
APP/PS1-TBI
Survival
Death
E
Elevated Plus Maze
%Time spent in open arms
D7 D10 1M 2M 3M 6M 7M 8M
Inj p=0.001 Geno p=0.073
Geno*Inj p=0.347
F
Elevated Plus Maze
%Time spent in closed arms
D7 D10 1M 2M 3M 6M 7M 8M
Inj p=0.001 Geno p=0.791
Geno*Inj p=0.624
G
RotaRod
Fall latency (s)
Baseline D1 D2 D7 D14 1M1 1M2 2M1 2M2 3M1 3M2 6M1 6M2 7M1 7M2 8M1 8M2
Inj p=0.100 Geno p=0.004
Geno*Inj p=0.374
WT-Sham
WT-TBI
APP/PS1-Sham
APP/PS1-TBI
