# Supplementary material for: CHIMERA repetitive mild traumatic brain injury induces chronic behavioural and neuropathological phenotypes in wild-type and APP/PS1 mice
Source: Alzheimers Res Ther. 2019 Jan 12;11:6. doi: 10.1186/s13195-018-0461-0 (PMC6330571; doi:10.1186/s13195-018-0461-0)
Supplement: Supplementary file 2 — Summary of p values. This table summarizes the p values of the experiments and assays reported in this study. (DOCX 23 kb) [file 13195_2018_461_MOESM2_ESM.docx]

**Additional file 7**: Summary of p-value table.

| **Experiment/Assay** | **Genotype** | **Injury** | **Genotype* Injury** | **Time** | **Genotype* Time** | **Injury* Time** | **Genotype* Injury* Time** |
| --- | --- | --- | --- | --- | --- | --- | --- |
| Passive Avoidance Duration | 0.192 | <0.001 | <0.001 | <0.001 | 0.995 | 0.263 | 0.838 |
| Passive Avoidance Initial Fear Memory | 0.522 | 0.026 | 0.061 | <0.001 | 0.578 | 0.174 | 0.417 |
| Passive Avoidance Rate of Extinction | 0.254 | 0.001 | 0.042 | 0.152 | 0.837 | 0.678 | 0.593 |
| Barnes Maze Acquisition Duration | <0.001 | <0.001 | 0.164 | <0.001 | 0.278 | 0.792 | 0.734 |
| Barnes Maze Cognitive Score | 0.004 | 0.008 | 0.263 | <0.001 | 0.842 | 0.783 | 0.07 |
| Barnes Maze Probe Trial - North Quadrant | 0.175 | 0.762 | 0.027 | 0.226 | 0.114 | 0.522 | 0.3 |
| Barnes Maze Probe Trial - Previous escape location | 0.019 | 0.575 | 0.084 | 0.013 | 0.44 | 0.299 | 0.833 |
| Barnes Maze Reverse Trial | <0.001 | 0.862 | 0.002 | 0.001 | 0.363 | 0.708 | 0.813 |
| Elevated Plus Maze Difference | 0.662 | <0.001 | 0.404 | 0.001 | 0.434 | 0.957 | 0.952 |
| Elevated Plus Maze Close | 0.073 | <0.001 | 0.347 | 0.001 | 0.783 | 0.604 | 0.989 |
| Elevated Plus Maze Open | 0.791 | 0.001 | 0.624 | <0.001 | 0.851 | 0.99 | 0.919 |
| Optic Tract Iba1 Density | 0.641 | <0.001 | 0.565 |  |  |  |  |
| Optic Tract Iba1 Size | 0.913 | <0.001 | 0.315 |  |  |  |  |
| Optic Tract Silver | 0.264 | 0.005 | 0.124 |  |  |  |  |
| Optic Tract GFAP | 0.518 | 0.007 | 0.253 |  |  |  |  |
| Optic Tract Neurofilament | 0.14 | 0.521 | 0.342 |  |  |  |  |
| Corpus Callosum Iba1 Density | 0.434 | 0.927 | 0.351 |  |  |  |  |
| Corpus Callosum Iba1 Size | 0.032 | 0.747 | 0.909 |  |  |  |  |
| Corpus Callosum Silver | 0.28 | 0.387 | 0.431 |  |  |  |  |
| Corpus Callosum GFAP | <0.001 | 0.661 | 0.334 |  |  |  |  |
| Prefrontal Cortex Iba1 Density | <0.001 | 0.487 | 0.51 |  |  |  |  |
| Prefrontal Cortex Iba1 Area | <0.001 | 0.934 | 0.509 |  |  |  |  |
| Prefrontal Cortex activated Iba1 Density | <0.001 | 0.335 | 0.299 |  |  |  |  |
| Prefrontal Cortex activated Iba1 Area | <0.001 | 0.688 | 0.673 |  |  |  |  |
| Prefrontal Cortex non-activated Iba1 Density | 0.146 | 0.337 | 0.681 |  |  |  |  |
| Prefrontal Cortex non-activated Iba1 Area | 0.002 | 0.527 | 0.472 |  |  |  |  |
| Prefrontal Cortex GFAP | <0.001 | 0.731 | 0.589 |  |  |  |  |
| Prefrontal Cortex DAPI | 0.912 | 0.492 | 0.103 |  |  |  |  |
| Amygdala Iba1 Density | 0.573 | 0.12 | 0.576 |  |  |  |  |
| Amygdala Iba1 Area | <0.001 | 0.009 | 0.08 |  |  |  |  |
| Amygdala activated Iba1 Density | <0.001 | 0.014 | 0.074 |  |  |  |  |
| Amygdala activated Iba1 Area | <0.001 | 0.003 | 0.01 |  |  |  |  |
| Amygdala non-activated Iba1 Density | 0.91 | 0.155 | 0.902 |  |  |  |  |
| Amygdala non-activated Iba1 Area | 0.394 | 0.146 | 0.68 |  |  |  |  |
| Amygdala GFAP | <0.001 | 0.455 | 0.432 |  |  |  |  |
| Amygdala DAPI | 0.614 | 0.228 | 0.012 |  |  |  |  |
| Hippocampus Iba1 Density | <0.001 | 0.14 | 0.06 |  |  |  |  |
| Hippocampus Iba1 Area | <0.001 | 0.009 | 0.08 |  |  |  |  |
| Hippocampus activated Iba1 Density | 0.014 | 0.194 | 0.303 |  |  |  |  |
| Hippocampus activated Iba1 Area | 0.017 | 0.154 | 0.251 |  |  |  |  |
| Hippocampus non-activated Iba1 Density | 0.001 | 0.145 | 0.061 |  |  |  |  |
| Hippocampus non-activated Iba1 Area | <0.001 | 0.12 | 0.048 |  |  |  |  |
| Hippocampus GFAP | <0.001 | 0.528 | 0.094 |  |  |  |  |
| Hippocampus DAPI | 0.714 | 0.574 | 0.352 |  |  |  |  |
| GuHCl-soluble A-beta40 |  | 0.662 |  |  |  |  |  |
| GuHCl-soluble A-beta42 |  | 0.593 |  |  |  |  |  |
| Carbonate-soluble A-beta40 |  | 0.504 |  |  |  |  |  |
| Carbonate-soluble A-beta42 |  | 0.854 |  |  |  |  |  |
| Carbonate-soluble Aducanumab-binding |  | 0.015 |  |  |  |  |  |
| Carbonate-soluble Human IgG1-binding |  | 0.959 |  |  |  |  |  |
| Carbonate-soluble Poly8029-binding |  | 0.175 |  |  |  |  |  |
| Loss of righting reflex | 0.851 | 0.361 | 0.917 | 0.018 | 0.978 | 0.832 | 0.739 |
| Neurological severity score | <0.001 | 0.173 | 0.835 | 0.036 | 0.132 | 0.607 | 0.936 |
| Mortality during ageing | 0.032 | 1 |  |  |  |  |  |
| Rotarod | 0.004 | 0.1 | 0.001 |  |  |  |  |
| Total tau | 0.024 | 0.145 | 0.88 |  |  |  |  |
| Phospho-Thr231 tau | <0.001 | 0.278 | 0.646 |  |  |  |  |
| Phospho:Total tau | <0.001 | 0.319 | 0.329 |  |  |  |  |
| Neurofilament-light | 0.207 | 0.51 | 0.297 |  |  |  |  |
| Parietal Cortex 6E10 |  | 0.056 |  |  |  |  |  |
| Prefrontal Cortex 6E10 |  | 0.743 |  |  |  |  |  |
| Amygdala 6E10 |  | 0.681 |  |  |  |  |  |
| Hippocampus 6E10 |  | 0.928 |  |  |  |  |  |
| Corpus Callosum 6E10 |  | 0.157 |  |  |  |  |  |
| Parietal Cortex ThioS |  | 0.761 |  |  |  |  |  |
| Prefrontal Cortex ThioS |  | 0.842 |  |  |  |  |  |
| Amygdala ThioS |  | 0.939 |  |  |  |  |  |
| Hippocampus ThioS |  | 0.364 |  |  |  |  |  |
| sAPP/GAPDH | <0.001 | 0.74 | 0.605 | - | - | - | - |
| APP-CTF/GAPDH | <0.001 | 0.34 | 0.803 |  |  |  |  |
