# Supplementary figures and images for: CHIMERA repetitive mild traumatic brain injury induces chronic behavioural and neuropathological phenotypes in wild-type and APP/PS1 mice
Source: Alzheimers Res Ther. 2019 Jan 12;11:6. doi: 10.1186/s13195-018-0461-0 (PMC6330571; doi:10.1186/s13195-018-0461-0)

## Corpus Callosum

**A**

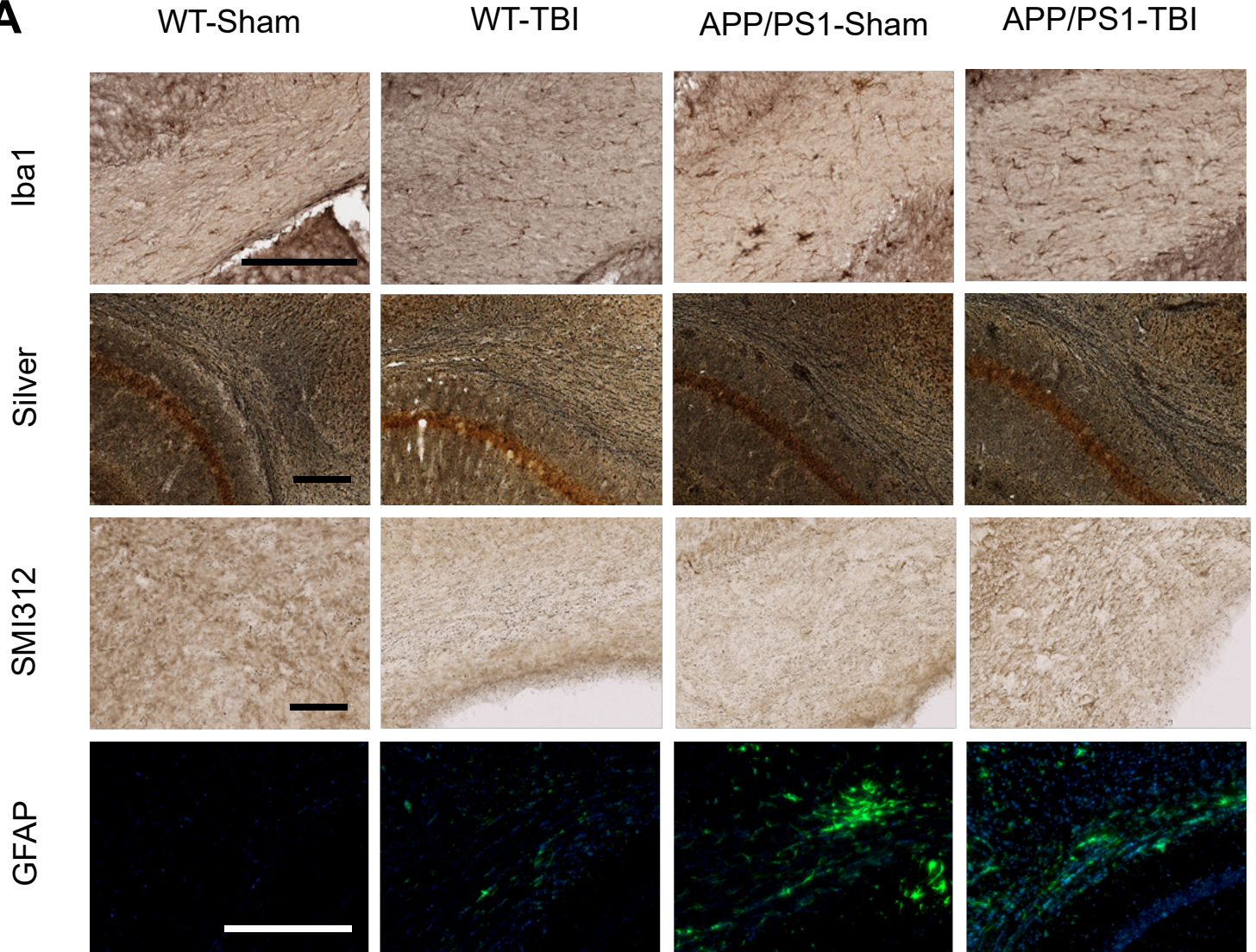

**B**

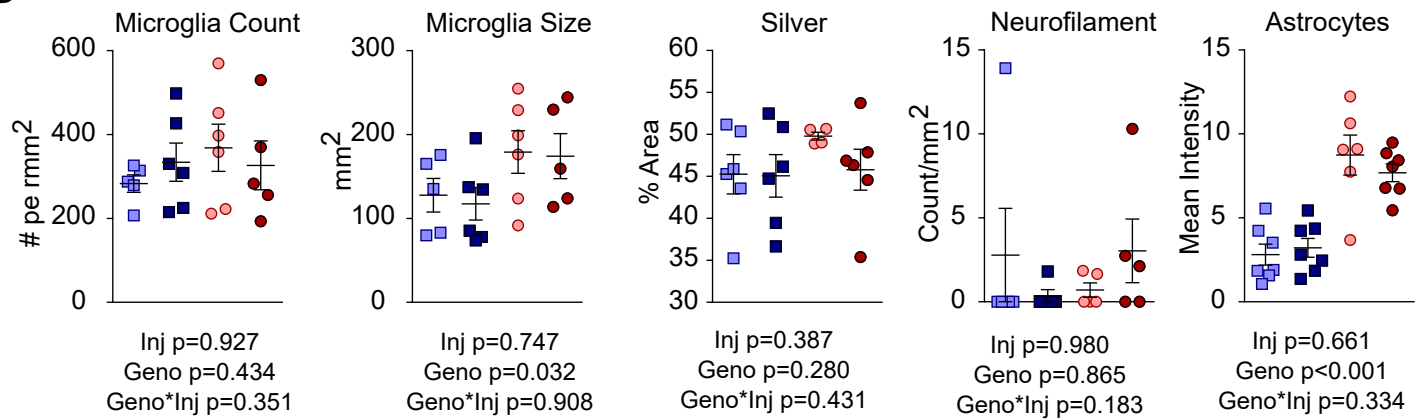

■ WT-Sham   
 ■ WT-TBI   
 ● APP/PS1-Sham   
 ● APP/PS1-TBI

Supplement: Supplementary file 3 — White matter pathology in the corpus callosum. a At 8 M post-TBI, histopathological analyses were performed on the corpus callosum at the level of the dorsal hippocampus, using Iba1 (microglia), NeuroSilver (axonal injury), SMI312A (neurofilament) and GFAP (astrocytes). Representative images for each stain are shown. (b) Quantification of (a) is plotted by reporting the density and size of microglia, the stain area of NeuroSilver, the density of neurofilament-positive axonal bulbs, and the GFAP immunofluorescence intensity of astrocytes. Scale bar represents 200 μm. Data are plotted as mean ± SE. (PDF 1515 kb) [file 13195_2018_461_MOESM3_ESM.pdf]

**A**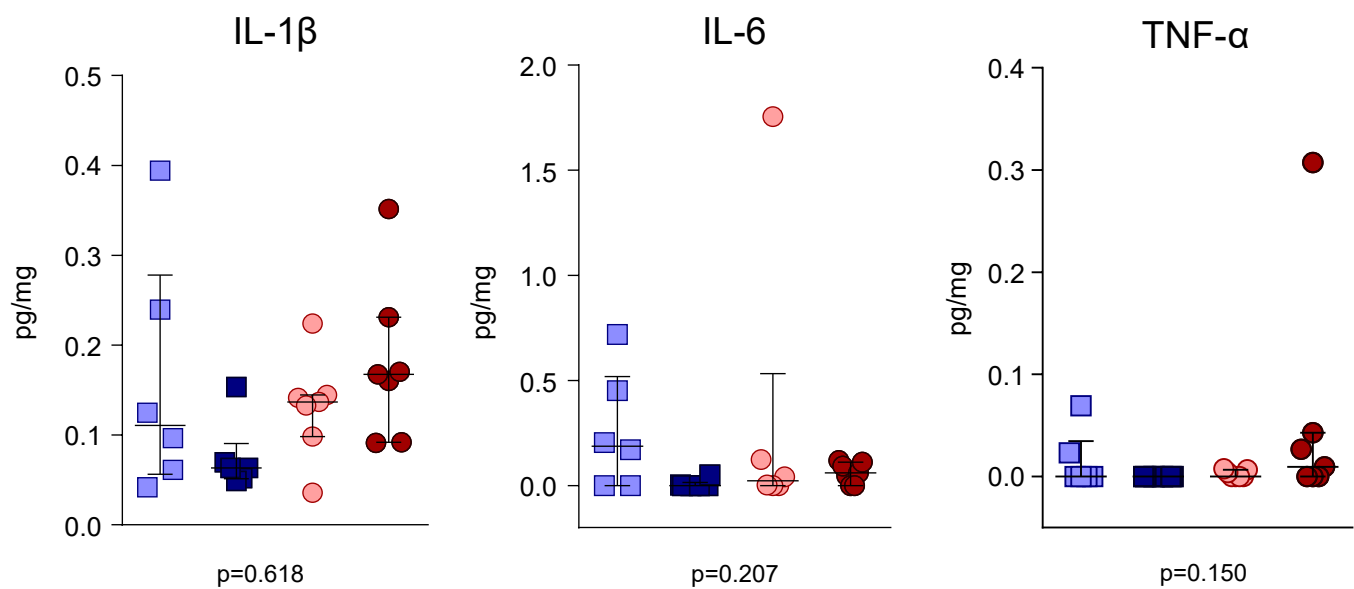**B**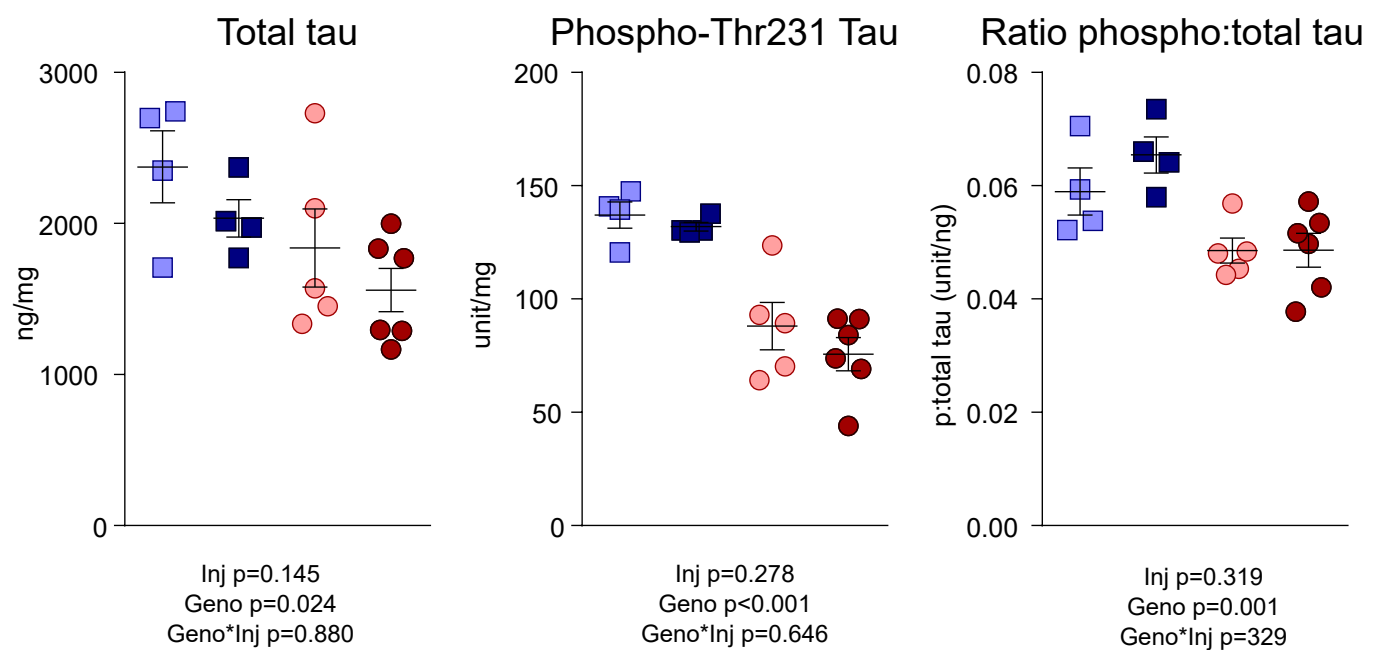**C**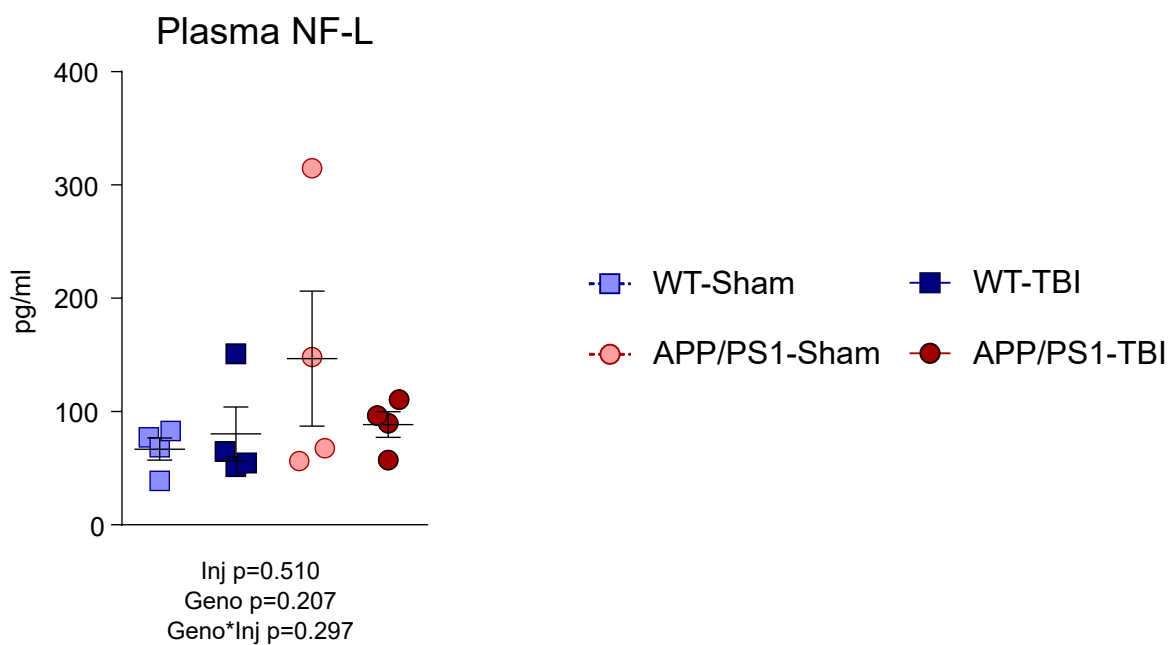

Supplement: Supplementary file 4 — Brain cytokines and tau and plasma neurofilament-light. The carbonate-soluble fraction of brain homogenates were assayed for (a) inflammatory cytokines including IL-1β, IL-6, and TNF-α and (b) total tau, p-tau at Thr231, and the ratio of p-tau to total tau. c Plasma levels of neurofilament-light at 8 M post-injury is also reported. In (a), data are plotted as median ± interquartile range. In (b) and (c), data are plotted as mean ± SE. (PDF 50 kb) [file 13195_2018_461_MOESM4_ESM.pdf]

**A**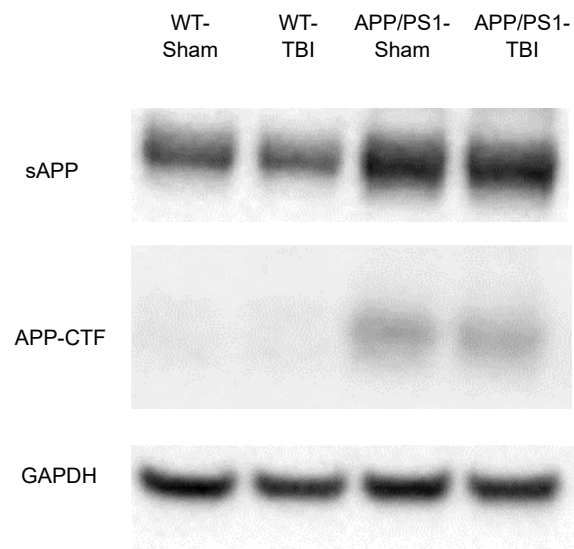**B**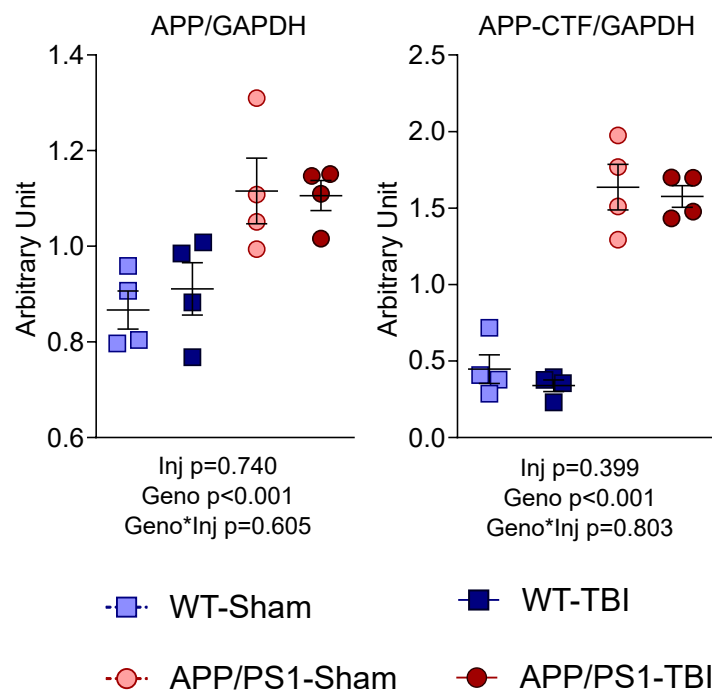

Supplement: Supplementary file 7 — Western blot analysis of Aβ metabolism. a Protein levels of soluble APP, APP-C-terminal fragment and GAPDH in carbonate-soluble brain homogenates were analysed by Western blotting. b Quantification of (a) by densitometry. Data are plotted as mean ± SE. (PDF 44 kb) [file 13195_2018_461_MOESM7_ESM.pdf]
