# Supplementary material for: CHIMERA repetitive mild traumatic brain injury induces chronic behavioural and neuropathological phenotypes in wild-type and APP/PS1 mice
Source: Alzheimers Res Ther. 2019 Jan 12;11:6. doi: 10.1186/s13195-018-0461-0 (PMC6330571; doi:10.1186/s13195-018-0461-0)
Supplement: Supplementary file 5 — Aβ deposits in grey and white matter. a 6E10 immunostaining for diffuse Aβ deposits was performed for the parietal cortex, fear and spatial memory-related areas, and the corpus callosum. The optic tract was immunonegative for 6E10 staining and thus not shown. b Quantification of (a) showing the percentage area stained by 6E10. Scale bar represents 100 μm. Data are plotted as mean ± SE. (PDF 1133 kb) [file 13195_2018_461_MOESM5_ESM.pdf]

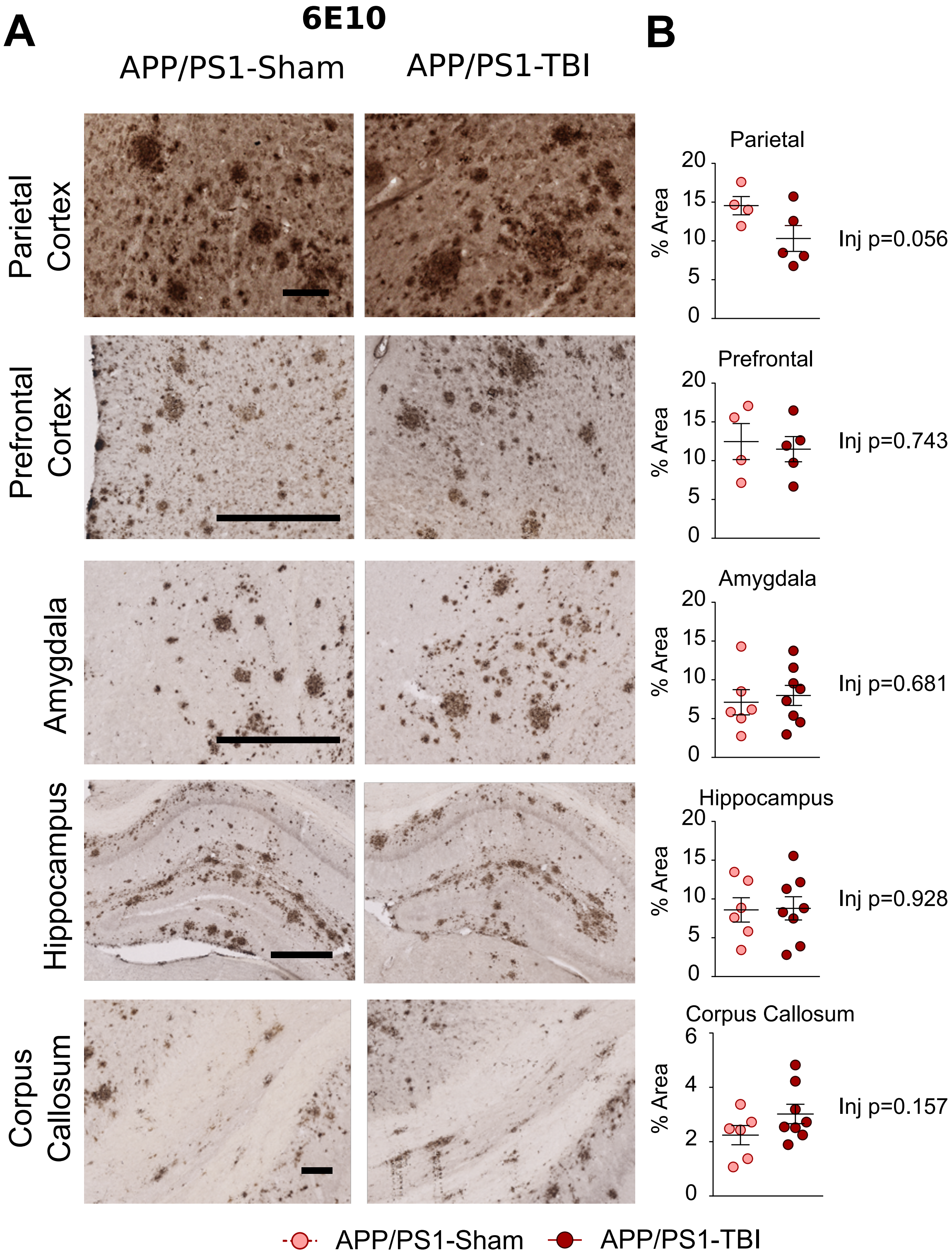
A
6E10
APP/PS1-Sham
APP/PS1-TBI
Parietal Cortex
Prefrontal Cortex
Amygdala
Hippocampus
Corpus Callosum
B
Parietal
Inj p=0.056
Prefrontal
Inj p=0.743
Amygdala
Inj p=0.681
Hippocampus
Inj p=0.928
Corpus Callosum
Inj p=0.157
% Area
APP/PS1-Sham
APP/PS1-TBI
