# Supplementary material for: CHIMERA repetitive mild traumatic brain injury induces chronic behavioural and neuropathological phenotypes in wild-type and APP/PS1 mice
Source: Alzheimers Res Ther. 2019 Jan 12;11:6. doi: 10.1186/s13195-018-0461-0 (PMC6330571; doi:10.1186/s13195-018-0461-0)
Supplement: Supplementary file 6 — Amyloid deposits in grey matter. a ThioS was used to stain fibrillary amyloid at the parietal cortex and in fear and spatial memory-related areas. b Quantification of (a) showing the percentage area stained by ThioS. Scale bar represents 100 μm. Data are plotted as mean ± SE. (PDF 151 kb) [file 13195_2018_461_MOESM6_ESM.pdf]

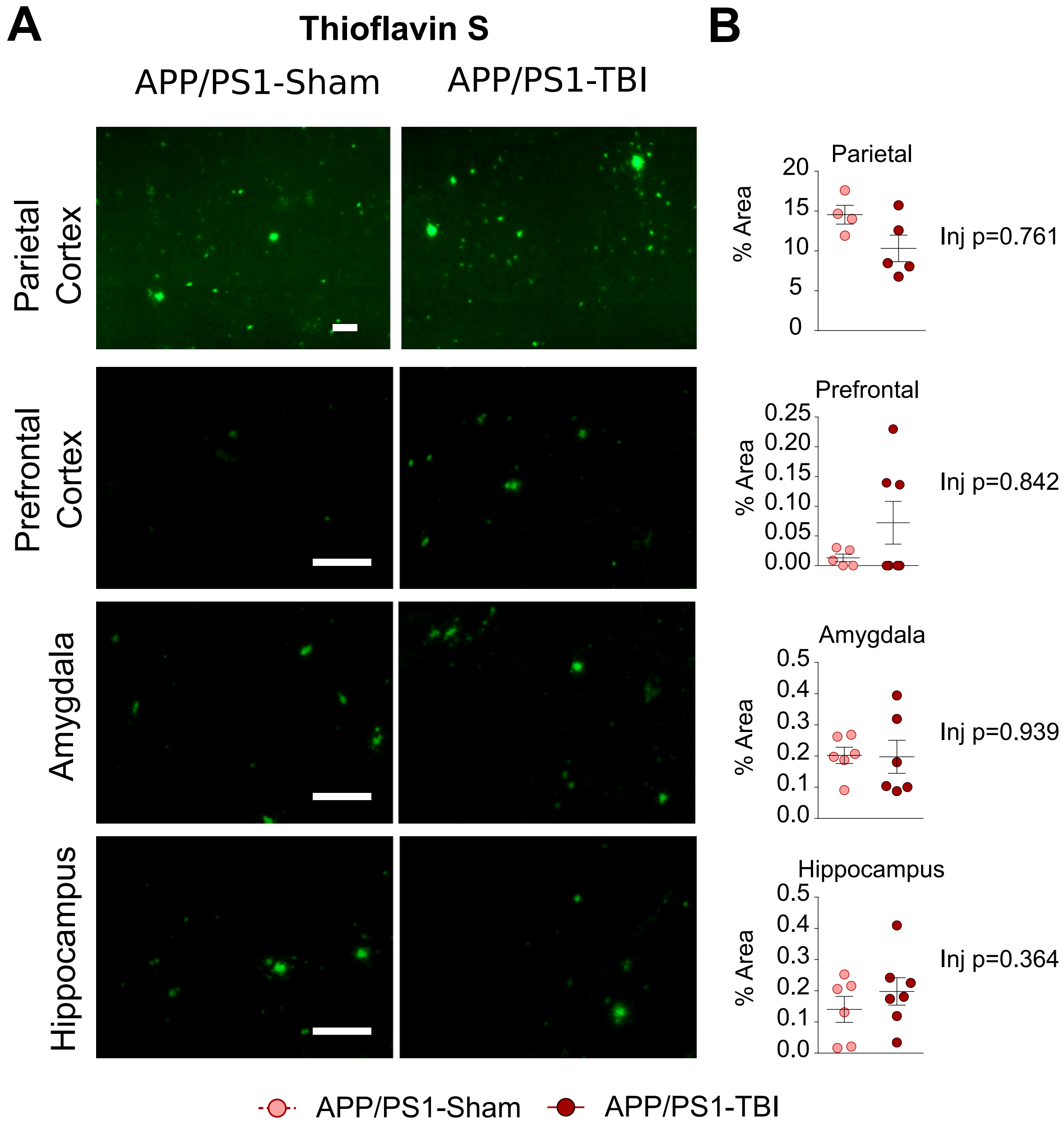
A
Thioflavin S
APP/PS1-Sham
APP/PS1-TBI
Parietal Cortex
Prefrontal Cortex
Amygdala
Hippocampus
B
Parietal
% Area
20
15
10
5
0
Inj p=0.761
Prefrontal
0.25
0.20
0.15
0.10
0.05
0.00
Inj p=0.842
Amygdala
0.5
0.4
0.3
0.2
0.1
0.0
Inj p=0.939
Hippocampus
Inj p=0.364
APP/PS1-Sham
APP/PS1-TBI
